# Prognostic prediction models for adverse birth outcomes: A systematic review

**DOI:** 10.7189/jogh.14.04214

**Published:** 2024-10-25

**Authors:** Achenef Asmamaw Muche, Likelesh Lemma Baruda, Clara Pons-Duran, Robera Olana Fite, Kassahun Alemu Gelaye, Alemayehu Worku Yalew, Lisanu Tadesse, Delayehu Bekele, Getachew Tolera, Grace J Chan, Yifru Berhan

**Affiliations:** 1 Health System and Reproductive Health Research Directorate, Ethiopian Public Health Institute, Addis Ababa, Ethiopia; 2 Department of Epidemiology and Biostatistics, Institute of Public Health, College of Medicine and Health Sciences, University of Gondar, Gondar, Ethiopia; 3 Maternal and Child Health Directorate, Federal Ministry of Health, Addis Ababa, Ethiopia; 4 Department of Epidemiology, Harvard T.H. Chan School of Public Health, Boston, Massachusetts, USA; 5 HaSET Maternal and Child Health Research Program, Addis Ababa, Ethiopia; 6 School of Public Health, Addis Ababa University, Addis Ababa, Ethiopia; 7 Department of Obstetrics and Gynaecology, Saint Paul’s Hospital Millennium Medical College, Addis Ababa, Ethiopia; 8 Deputy Director General Office for Research and Technology Transfer Directorate, Ethiopian Public Health Institute, Addis Ababa, Ethiopia; 9 Department of Paediatrics, Boston Children’s Hospital, Harvard Medical School, Boston, Massachusetts, USA

## Abstract

**Background:**

Despite progress in reducing maternal and child mortality worldwide, adverse birth outcomes such as preterm birth, low birth weight (LBW), small for gestational age (SGA), and stillbirth continue to be a major global health challenge. Developing a prediction model for adverse birth outcomes allows for early risk detection and prevention strategies. In this systematic review, we aimed to assess the performance of existing prediction models for adverse birth outcomes and provide a comprehensive summary of their findings.

**Methods:**

We used the Population, Index prediction model, Comparator, Outcome, Timing, and Setting (PICOTS) approach to retrieve published studies from PubMed/MEDLINE, Scopus, CINAHL, Web of Science, African Journals Online, EMBASE, and Cochrane Library. We used WorldCat, Google, and Google Scholar to find the grey literature. We retrieved data before 1 March 2022. Data were extracted using CHecklist for Critical Appraisal and Data Extraction for Systematic Reviews of Prediction Modelling Studies. We assessed the risk of bias with the Prediction Model Risk of Bias Assessment tool. We descriptively reported the results in tables and graphs.

**Results:**

We included 115 prediction models with the following outcomes: composite adverse birth outcomes (n = 6), LBW (n = 17), SGA (n = 23), preterm birth (n = 71), and stillbirth (n = 9). The sample sizes ranged from composite adverse birth outcomes (n = 32–549), LBW (n = 97–27 233), SGA (n = 41–116 070), preterm birth (n = 31–15 883 784), and stillbirth (n = 180–76 629). Only nine studies were conducted on low- and middle-income countries. 10 studies were externally validated. Risk of bias varied across studies, in which high risk of bias was reported on prediction models for SGA (26.1%), stillbirth (77.8%), preterm birth (31%), LBW (23.5%), and composite adverse birth outcome (33.3%). The area under the receiver operating characteristics curve (AUROC) was the most used metric to describe model performance. The AUROC ranged from 0.51 to 0.83 in studies that reported predictive performance for preterm birth. The AUROC for predicting SGA, LBW, and stillbirth varied from 0.54 to 0.81, 0.60 to 0.84, and 0.65 to 0.72, respectively. Maternal clinical features were the most utilised prognostic markers for preterm and LBW prediction, while uterine artery pulsatility index was used for stillbirth and SGA prediction.

**Conclusions:**

A varied prognostic factors and heterogeneity between studies were found to predict adverse birth outcomes. Prediction models using consistent prognostic factors, external validation, and adaptation of future risk prediction models for adverse birth outcomes was recommended at different settings.

**Registration:**

PROSPERO CRD42021281725.

Although maternal and child health (MCH) programmes have made progress in recent years all over the world, many regions of the world continue to experience persistently high rates, or progress is now stagnating of adverse birth outcomes such as stillbirth, preterm birth, and low birth weight (LBW) [1-3]. Globally, adverse birth outcomes affect millions of newborns. Low- and middle-income countries (LMICs) account for 98% of all stillbirths, with three-quarters of these occurring in sub-Saharan Africa, where women face socioeconomic challenges and often lack access to maternity services [4,5]. The burden of adverse birth outcomes is increasing around the world [6]. LBW is strongly associated with perinatal death [7].

Addressing MCH issues during the perinatal period is critical for the health of mothers and neonates. Continuum of maternity care (CMC) provides a window of opportunity to screen mothers to prevent and manage adverse birth outcomes [8]. To provide effective and efficient CMC, data-driven health care approaches have been identified, and prognostic prediction models are becoming more popular [9,10]. Models that predict adverse birth outcomes have been introduced to reduce foetal and neonatal mortality [11,12]. Developing accurate risk prediction models allows the estimation of pregnancy-related risks through risk stratification and identifying women and babies at higher risk. The absolute risk of complications can then be calculated and used to help develop personalised care models [13]. However, many risk prediction models are not used in clinical practices due to poor predictive performance [14,15].

Despite these challenges, the potential benefit of risk detection approaches, such as using regression formula models, score chart rules, or nomograms to improve the delivery of high-quality interventions, may be substantial [16]. A suitable and effective prognostic prediction model can calculate the absolute risk of adverse birth outcomes based on unique individual characteristics such as social demographics, maternal factors, obstetric history, and clinical biomarkers. However, most prognostic models investigated to help explain the large variation in patient prognosis produced contradictory results from studies of varying quality and poor predictive performance [14]. Knowing the gaps in common predictive models used in Ethiopia could help to predict adverse birth outcomes. With this backdrop, we aimed to review the existing prognostic prediction models for adverse birth outcomes, qualitatively describe their characteristics, and quantitatively compare their performance.

## METHODS

The protocol was registered on PROSPERO (CRD42021281725). To present the results, we used the 2020 Preferred Reporting Items for Systematic Review and Metanalysis (PRISMA) checklist [17].

### Study outcomes

We summarised global prediction models that focused on at least one of four adverse birth outcomes: 1) stillbirth, defined as the death of a foetus after 28 weeks of gestation and/or weighing at least 1000 g in low-income countries and after 20 weeks of gestation and/or weighing at least 500 g in high-income countries [18], 2) preterm birth, defined as giving birth before 37 weeks of gestation [19], 3) LBW, defined as a birth weight of a neonate below 2500 g [20], and 4) small for gestational age (SGA), defined as birth weight <10th percentile for sex and gestational age [21].

### Eligible studies

We used the Population, Index prediction model, Comparator, Outcome, Timing, and Setting (PICOTS) approach to declare inclusion and exclusion criteria: P (pregnant women), I (index prediction models for adverse birth outcomes), C (not applicable), O (adverse birth outcomes), T (prediction of adverse birth outcomes during pregnancy), and S (risk stratification for adverse birth outcomes in the clinical set up). Studies were eligible for inclusion if published in peer-reviewed journals or grey literature. This review used prediction models from cohort, nested case-control, case-cohort, and randomised control trials. We excluded case reports, reviews, and letters that did not address the prediction model for adverse birth outcomes, as well as protocols and studies whose entire texts were unavailable in English.

### Search strategy

We retrieved articles published before 1 March 2022 from PubMed/ MEDLINE, Scopus, CINAHL, Web of Science, African Journals Online, EMBASE, and the Cochrane Library. We searched for grey literature such as reports, evaluations, and guidelines from government, international organisations, conference presentations or preprints, using WorldCat, Google and/or Google Scholar. We created search strategies for each database using Medical Subject Headings terms for the four identified adverse birth outcomes, plus the terms potential predictors and prediction models, which were used separately and in combination with the Boolean operators ‘OR’ and ‘AND’ to broaden or narrow the search as needed. There was also a mix of expanded search terms and free-text searches. The reference lists of the retrieved studies were then accessed to identify additional articles and screen them for eligibility for this review. Two researchers, AAM and LLB, conducted the searches concurrently. We also included a methodological filter for qualitative studies (Table S1 in the **Online Supplementary Document**).

### Study selection

Each study was evaluated against a predefined eligibility criterion to determine whether it would be included in the systematic review. Two independent reviewers (AAM and LLB) performed title and abstract screening, which was followed by screening the full text. Any disagreements between the two reviewers were resolved through discussion or by a third reviewer (YBM). After eliminating duplicated articles, the eligible articles were imported into EndNote, version 20 (Clarivate Analytics, Philadelphia, Pennsylvania, USA) from each database and search engines/repositories.

### Assessment of risk of bias

We used the Prediction Model Risk of Bias Assessment (PROBAST) tool to assess how the participants were selected, the predictors and outcomes were identified, and the analyses were conducted. The tool has four key domains (participants, predictors, outcome, and analysis) structured in 20 signalling questions to facilitate risk-of-bias assessment. Each domain was rated as having a high, low, or unclear risk of bias [22].

We also selected the prediction model utilised in the final analysis and provided a rationale. The implemented method’s strengths and drawbacks, as well as how PROBAST communicated to estimate the individual risk of the outcomes, that is, risk ratios (RR) and hazard ratios (HR), were retrieved. We also evaluated the model’s internal and/or external validation. Similarly, data on model discrimination from the area under the receiver operating characteristics curve (AUROC) or C-statistic were evaluated.

Each study’s quality and reliability were determined by the following factors: study design, sample size, analytic procedures, and missing data. The predictability of the models’ predicted parameters, as well as the research findings, were evaluated. The number of predictive components used in the model, as well as whether internal and external model validation was performed, was determined by the analysis quality. We also included a quality summary because low-quality studies may not have used the most effective statistical approaches [23]. Similarly, for lower-quality research, any conclusions about outcomes and performance metrics from included studies were treated with caution.

### Data extraction

Data extraction was done by two independent reviewers (AAM and ROF). Inconsistencies were resolved by a tiebreaker reviewer (YBM). The Checklist for Critical Appraisal and Data Extraction For Systematic Reviews of Prediction Modelling Studies (CHARMS) was used [24]. We extracted the first author’s name, study year, sample size, country, study design, length of follow-up, predictors, outcomes, models, and model-related issue were extracted.

### Analysis and data presentation

Preferred Reporting Items for Systematic Reviews and Meta-Analyses (PRISMA) 2020 flow diagram and Cochrane Handbook for Systematic Reviews guided the review used to report and present the results [18,25]. The recommendations from the Transparent Reporting of a Multivariable Prediction Model for Individual Prognosis or Diagnosis (TRIPOD) statement were also employed [19]. A narrative summary of results from the external validation, AUROC, and calibration was presented for each study. The performance of the model was evaluated using the value of AUROC≤0.5, which suggested no discrimination ability; 0.5<AUROC<0.7 was considered indicative of poor discrimination, 0.7≤AUROC<0.8 indicated good discrimination, 0.8≤AUROC<0.9 indicated excellent discrimination and AUROC≥0.9 was considered indicative of outstanding discrimination performance. Data were presented in summary tables and, where applicable, graphically.

## RESULTS

### Study selection

**Figure 1:**
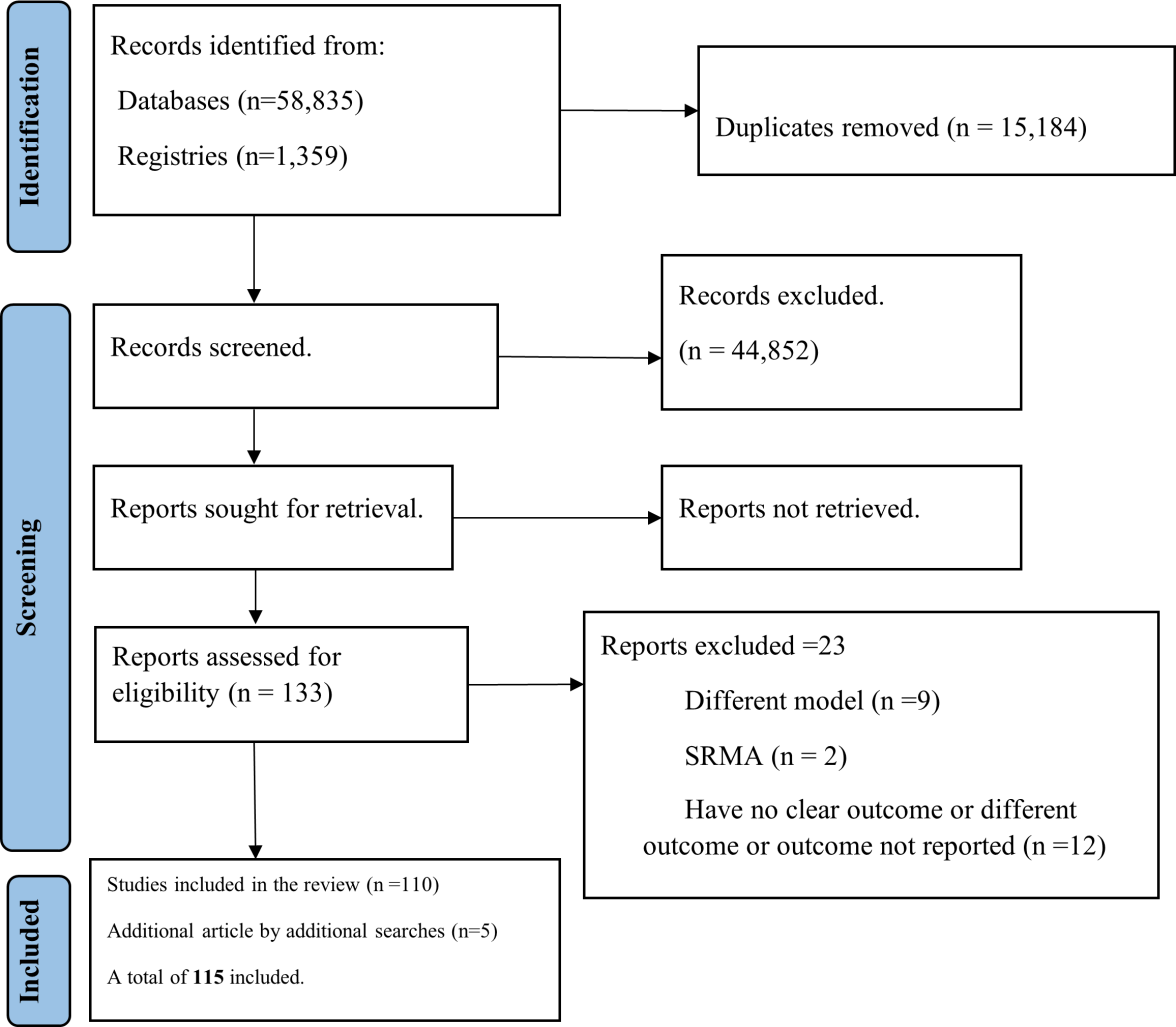
PRISMA flow diagram for the inclusion and exclusion criteria of a systematic review, 2022.

We identified 60 194 studies, 58 835 through database searches and 1359 through reference (snowball) searching. After removing duplicates, 45 010 studies were selected for title, abstract, and full-text screening. Of these, 158 studies were selected for full-text assessment. We excluded 48 articles due to lack of a prediction model, diagnostic prediction, systematic review/ meta-analysis, or no preterm, stillbirth, SGA or LBW prediction outcome. Additionally, six articles were found by reference (snowball) searching. Finally, 115 studies were included in this systematic review (**Figure 1**).

### Study characteristics

Of the 115 prediction models identified, we categorised them as composite adverse birth outcomes (n = 6), LBW (n = 17), SGA (n = 23), preterm birth (n = 71), and stillbirth (n = 9). Only nine studies were conducted on LMICs. The sample size for the studies ranged between 31 and 448 852. 24 articles indicated how missing data was handled, including multiple imputation, complete case analysis, and single regression imputation. In 74.8% (n = 86/115) of the studies, the presence and handling of missing data were frequently omitted from analysis (**Table 1**). Model performance was mostly judged by AUROC, specificity, sensitivity, positive predictive value, and negative predictive value. The detailed study characteristics prediction models for each adverse outcome are presented in Tables S2–6 in the **Online Supplementary Document**.

**Table 1:** Summary of study characteristics prediction models for each adverse birth outcomes of a systematic review, 2022

Outcomes	Included studies (n)	Sample size, n (min/max)	Studies by geographic region, n (developed/LMICs)	Externally validated studies (n)
Preterm birth	71	31 / 15 883 784	68 / 3	7
LBW	17	97 / 27 233	12 / 5	0
Small for gestational age	23	41 / 116 070	23 / 0	1
Stillbirth	9	180 / 76 629	9 / 0	1
Composite adverse birth outcome	6	31 / 549	5 / 1	1

LBW – low birth weight, LMICs – low- and middle-income countries, max – maximum, min – minimum

### Risk of bias and concerns regarding the applicability of models

**Figure 2:**
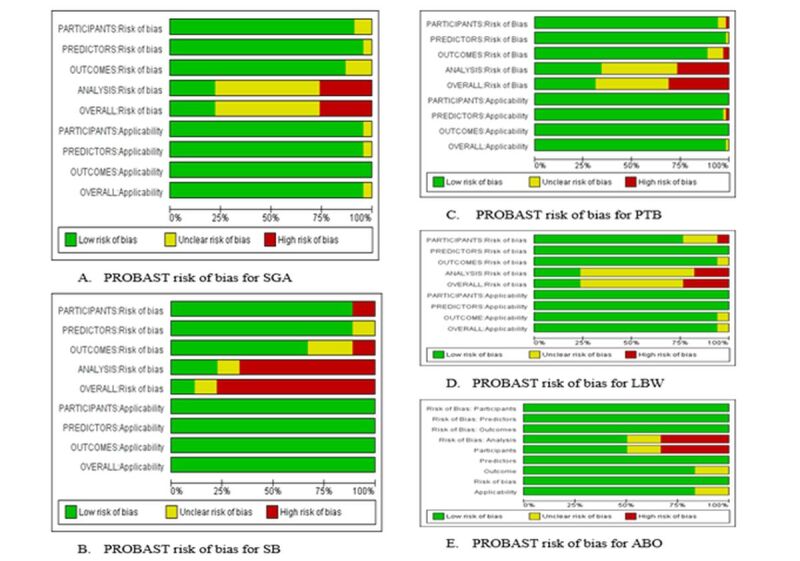
Systematic review using the Prediction Model Risk of Bias Assessment tool for predicting adverse birth outcomes of a systematic review, 2022.

The risk assessment outcomes differed among studies and by examined items. Overall, participant selection was deemed low risk. However, an overall high risk of bias was reported on prediction models for SGA (26.1%), stillbirth (77.8%), preterm birth (31%), LBW (23.5%), and composite adverse birth outcome (33.3%). The main reason for the high risk of analysis bias was a lack of reporting in the methods section. For instance, no reporting of internal validation was addressed when selecting an optimal model among several candidate prediction models. Some studies failed to incorporate the final model equation. There was considerable concern about the relevance of the three studies to the systematic review question and predictors applicability (**Figure 2**).

### Comparison of model development and predictive performance

The AUROC was the most used metric to describe model performance for each adverse birth outcome. Most studies used univariate analysis with a predetermined *P*-value to select candidate predictors for inclusion in the model. Studies stated model derivation, external validation, or incremental value (n = 90/115), external validation (n = 10/115), impact study (n = 3/115), and incremental value (n = 11/115). The prediction models in most articles were developed using logistic regression and survival modelling. Most used the stepwise forward selection method for predictor selection during multivariable modelling, and some used the lasso regression approach for model derivation.

The discrimination of prediction models was reported using the C-statistic or the AUROC, as well as some other calibrations (n = 16/115). 65.2% (n = 75/115) of the studies included classification measures. When model development and performance evaluation use the same data set, prediction model performance is overestimated; this was the case for all of the studies except the two that used an external data set for validation.

The model formula with regression coefficients, score chart, and prediction rules were used for ease of use and clinical application in 115 studies. Nomograms, on the other hand, are rarely used. From the 71 studies focused on preterm prediction models, the preterm rates differed depending on whether high-risk pregnant women were included and the outcome definition used. Thirty studies (42.3%) have an AUROC for prognostic prediction of preterm birth, and fourteen have good model discrimination performance (AUROC>0.7). The AUROC for the prediction of preterm birth ranges from 0.51 to 0.83.

Of the 23 studies on the prognostic prediction of SGA, 16 studies (69.6%) had good model discrimination performance (AUROC>0.7). The summary AUROC had good discrimination performance with a prediction interval from 0.54 to 0.81. Similarly, among the 17 studies for the prognostic prediction of LBW-reported AUROC, only one study has poor model discrimination performance. The AUROC for prediction of LBW ranged from 0.60 to 0.84. Among the nine studies that reported AUROC for the prognostic prediction of stillbirth, four had good model discrimination performance (AUROC>0.7). The AUROC for the prediction of stillbirth ranged from 0.65 to 0.72.

### Distribution of prognostic factors

**Figure 3:**
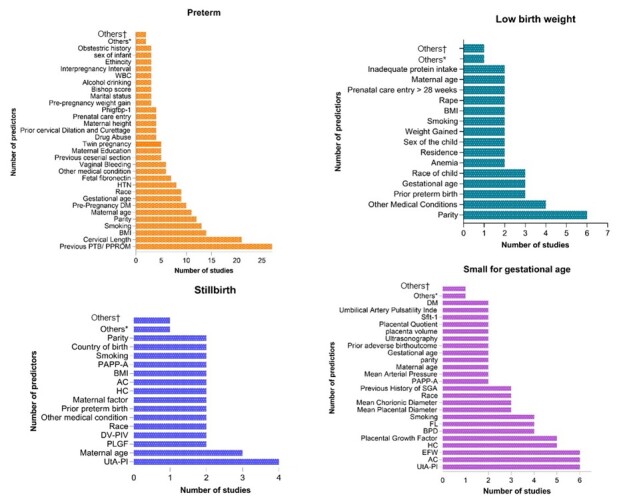
Distribution of prognostic factors across adverse birth outcomes of a systematic review, 2022. *Other prognostic factors included two times. †Other prognostic factors included one time.

We assessed the type of prognostic factors included in each adverse pregnancy outcome. Different prognostic factors were included for preterm, LBW, stillbirth, and SGA prediction models. Clinical characteristics and biomarkers were included for each outcome prediction. Maternal characteristics were the most widely used prognostic factors for preterm birth prediction. Prior, preterm birth, cervical length, body mass index (BMI), smoking history, parity, and maternal age were the top prognostic factors. Parity, mother’s medical condition, prior preterm births, and race were prognostic factors for LBW prediction. Furthermore, the uterine artery pulsatility index (UtA-PI) was the top-used prognostic factor for stillbirth and SGA prediction. The major predictors listed were maternal characteristics, advanced maternal age, parity, pre-pregnancy weight, BMI, maternal characteristics and cervical length, maternal characteristics with biomarkers pregnancy-associated plasma protein A (PAPP-A), placental growth factor (PLGF), UtA-PI, placental volume (PV), and pregnancy-related complication (previous history of hypertension, maternal characteristics with biomarkers and cervical length) (**Figure 3**).

## DISCUSSION

In this systematic review on prediction models for adverse birth outcomes, our methodological quality assessment revealed various shortcomings on the model development. We identified insufficient sample size, poor management of missing data, and a lack of internal validation methods. As a result, the reviewed models were of moderate to low quality. A high risk of bias was reported in each type of adverse birth outcome. Mainly more than three-fourths of a high risk of bias was observed in stillbirth. The main reason for the high risk of analysis bias was a lack of reporting in the methods section. For instance, no reporting of internal validation was addressed when selecting an optimal model among several candidate prediction models.

All prognostic prediction models have the same goal: to estimate an individual's unique risk of a specific event occurring in the future using prognostic determinants [20,21,26]. The domain of pure prediction is anti-parsimonious [27]; many possible elements can generate more accurate predictions for specific occurrences when integrated in complicated, nonlinear ways [28]. Specific predictors can be incorporated into prediction models of adverse birth outcomes based on routinely accessible clinical features, to direct screening and/or primary preventive initiatives.

However, the shortcomings identified all likely lead to overfitted prediction models, making it less likely for a model to function effectively in practice, whether in the same or a different population. Importantly, the likelihood of overfitting is considerable because most authors did not disclose the number of potential predictors considered or the predictor selection technique utilised. Poor missing data management can also be a source of bias. Only a few studies addressed missing data in accordance with current standards [29-34]. Furthermore, as a critical step before implementation, the built prediction models must be validated in external data sets.

Some of the prediction models were successfully developed and have internally validated, basic and extended models that could predict the risk of developing adverse birth outcome. The AUC also showed a good discrimination of the model’s performance in predicting each in predicting each adverse birth outcome [35-41]. However, these prediction models on the adverse birth outcomes require external validation before they can be used with confidence in clinical practice, as validation is a critical step to ensure that models perform similarly in new populations.

For some of the prediction models, the nomogram showed good calibration for predicting the likelihood of adverse birth outcomes. This suggests that preventive approaches and focused care would be consistent with a larger trend towards a more personalised approach to health care delivery: ‘the right treatment for the right person at the right time’ [42]. A prognostic prediction model for adverse birth outcomes that is intended to facilitate clinical decision-making throughout pregnancy would ideally incorporate clinically important and patient-aligned outcomes, including pregnancy complications impacting the mother and foetus [43,44]

Overall, a wide range of prognostic indicators were utilised to predict adverse birth outcomes. This systematic review revealed prior preterm birth, cervical length, body mass index, parity, and advanced maternal age were the most commonly utilised predictive factors for preterm. Poor maternal conditions were predictors for LBW prediction, while the uterine artery pulsatility index was the most commonly utilised prognostic factor for stillbirth and SGA prediction. Interestingly, maternal characteristics were included in mot prediction models with additional biomarkers (PAPP-A, PLGF, UtA-PI, PV), which had potential implications for improving the early detection of adverse birth outcomes. A risk strategy has been used in developing countries to identify high-risk pregnant women for adverse events. In contrast, risk factors are frequently non-medical and poor predictors of maternal risks [45]. It is not possible to anticipate or avoid adverse birth outcomes by using characteristics such as age, parity, and booking status. Furthermore, the risk approach relies on basic heuristics (relying on experience to diagnose patients, resulting in variations in service quality) [46]. Certain health care decisions necessitate a rigorous approach to deliver ideal patient care while also utilising a more accurate risk prediction model for adverse birth outcomes. This review suggests that predictive models could add value to maternity services by preventing adverse birth outcomes.

Hence, the current systematic review examined the predictive accuracy of models for adverse birth outcomes. Based on the available evidence, this review would provide recommendations by focusing on maternal characteristics and biomarkers for prognostic prediction models, as well as best practices for quantitatively summarising the model’s predictive performance using the easily available predictors at the different settings.

### Strengths and limitations

This review provides insight into the risk of adverse birth outcomes in routine clinical practice based on a validated search strategy for prediction models. The CHARMS guidelines and the PROBAST tool are used to thoroughly assess the quality of all studies. However, biomarkers studied for predicting adverse birth outcomes showed limited predictive performance.

## CONCLUSIONS

This review provided an overview of prognostic models for adverse birth outcomes. Overall, a wide range of prognostic indicators were utilised to predict adverse birth outcomes. By far, several widely varying models for predicting adverse birth outcomes have been developed, with some yielding promising results and having modest predictive performance. The high heterogeneity between studies and the potential of bias makes it difficult to identify the best model or conduct an aggregated analysis of prognostic models. The area under the AUROC curve was the most used metric to describe model performance for each adverse birth outcome.

This systematic review revealed that maternal clinical features were the most utilised prognostic markers for preterm and low birth weight prediction, while UtA-PI was used for stillbirth and small for gestational age prediction. We recommended the more accurate risk prediction for adverse birth outcomes may be possible if common risk factors are combined with biomarkers. Although most studies showed promising performance of prognostic prediction models, this systematic review reveals that the majority have not been externally evaluated. We recommend that the emphasis be shifted toward external validation at different time periods and areas and consecutive adaption of the future risk prediction models for adverse birth outcomes or that simplified models be provided that can be used in different settings. Furthermore, a summary input on the clinical utility of the prediction model would be incorporated into the existing programme for implementation in health care and beyond, integrating risk prediction to generate personalised approaches to public health interventions.

**Acknowledgements:** We appreciate the Ethiopian Public Health Institute, Saint Paul's Hospital Millennium Medical College, and Harvard T.H. Chan School of Public Health for their support and commitment to our work. We acknowledge all authors of studies included in this review. We appreciate Sofonyas Abebaw Truneh (PhD fellow at Monash University) for his professional assistance (risk of bias analysis).

## Additional material


Online Supplementary Document
