# Supplementary material for: Prognostic prediction models for adverse birth outcomes: A systematic review
Source: J Glob Health. 2024 Oct 25;14:04214. doi: 10.7189/jogh.14.04214 (PMC11503507; doi:10.7189/jogh.14.04214)
Supplement: Online Supplementary Document [file jogh-14-04214-s001.pdf]

Table S1: Search strategy

Data base	Search strategies	Result
	**PubMed**	
#1	((Pregnant women[MeSH Terms])) OR (Pregnant women[Title/Abstract])) OR (Pregnant woman[MeSH Terms])) OR (Pregnant woman[Title/Abstract])) OR (Pregnancy[MeSH Terms])) OR (Pregnancy[Title/Abstract])) OR (newborn[MeSH Terms])) OR (newborn*[Title/Abstract])) OR (neonatal[MeSH Terms])) OR (neonatal[Title/Abstract])) OR (gestation[MeSH Terms])) OR (gestation[Title/Abstract])) OR (women[MeSH Terms])) OR (women[Title/Abstract])) OR (mother[MeSH Terms])) OR (mother*[Title/Abstract])) OR (female[MeSH Terms])) OR (female[Title/Abstract])) OR (Pregnant mother[MeSH Terms])) OR (Pregnant mother*[Title/Abstract])) OR (antenatal mothers[MeSH Terms])) OR (antenatal mother*[Title/Abstract])) OR (antepartum women[MeSH Terms])) OR (antepartum women[Title/Abstract])) OR (neonate[MeSH Terms])) OR (neonate*[Title/Abstract])) OR (baby[MeSH Terms])) OR (baby[Title/Abstract])) OR (child*[MeSH Terms])) OR (child*[Title/Abstract])) OR (infant[MeSH Terms])) OR (infant*[Title/Abstract])) OR (infancy[MeSH Terms])) OR (infancy[Title/Abstract])	
#2	(((risk[MeSH Terms])) OR (risk*[Title/Abstract])) OR (probability[MeSH Terms])) OR (probabilit*[Title/Abstract])) OR (causality[MeSH Terms])) OR (causality[Title/Abstract])) OR (causation[MeSH Terms])) OR (causation[Title/Abstract])) OR (enabling factor[MeSH Terms])) OR (enabling factor*[Title/Abstract])) OR (predisposing factor[MeSH Terms])) OR (predisposing factor*[Title/Abstract])) OR (prognostic factor[MeSH Terms])) OR (prognostic factor*[Title/Abstract])) OR (prognostic determinant[MeSH Terms])) OR (prognostic determinant* [Title/Abstract])) OR (determinant[MeSH Terms])) OR (determinant*[Title/Abstract])) OR (factor[MeSH Terms])) OR (factor*[Title/Abstract])) OR (predictor[MeSH Terms])) OR (predictor*[Title/Abstract])) OR (risk factor[MeSH Terms])) OR (risk factor*[Title/Abstract])	
#3	(((prediction model[MeSH Terms]) OR (prediction model*[Title/Abstract])) OR (prediction[MeSH Terms])) OR (prediction[Title/Abstract])) OR (predictive[MeSH Terms])) OR (predictive[Title/Abstract])) OR (forecasting[MeSH Terms])) OR (forecasting[Title/Abstract])) OR (prognostic model[MeSH Terms])) OR (prognostic model*[Title/Abstract])) OR (predictive model[MeSH Terms])) OR (predictive model*[Title/Abstract])	
#4	((((((((((((((((((((((((((((((((((stillbirth[MeSH Terms]) OR (stillbirth*[Title/Abstract])) OR (intrauterine fetal death[MeSH Terms])) OR (intrauterine fetal death[Title/Abstract])) OR (preterm[MeSH Terms])) OR (preterm[Title/Abstract])) OR (preterm birth[MeSH Terms])) OR (preterm birth*[Title/Abstract])) OR (premature[MeSH Terms])) OR (premature*[Title/Abstract])) OR (Premature Birth[MeSH Terms])) OR (Premature Birth*[Title/Abstract])) OR (preterm labor[MeSH Terms])) OR (preterm labor[Title/Abstract])) OR ("low birth weight"[MeSH Terms])) OR ("low birth weight"[Title/Abstract])) OR ("low birthweight"[MeSH Terms])) OR ("low birthweight"[Title/Abstract])) OR (low birth weight[MeSH Terms])) OR (low birth weight[Title/Abstract])) OR (LBW[MeSH Terms])) OR (LBW[Title/Abstract])) OR ("small-for-gestational age"[MeSH Terms])) OR ("small-for-gestational age"[Title/Abstract])) OR ("small for gestational age"[MeSH Terms])) OR ("small for gestational age"[Title/Abstract])) OR (SGA[MeSH Terms])) OR (SGA[Title/Abstract])) OR ("fetal growth restriction"[MeSH Terms])) OR ("fetal growth restriction"[Title/Abstract])) OR (Preterm delivery[MeSH Terms]) OR (Preterm delivery[Title/Abstract])) OR (Prematurity[MeSH Terms])) OR (Prematurity[Title/Abstract])) OR (premature labor[MeSH Terms])) OR (premature labor[Title/Abstract])) OR (Fetal death[MeSH Terms])) OR (Fetal death[Title/Abstract])	

#5	#1 AND #2 AND #3 AND#4<br>Filter by study type, language, Text availability (Abstract, Free full text, Full text)	2,842<br>February 25, 2022<br>08:19:47PM
	Embase	
#1	pregnancy:ti,ab,kw OR 'pregnancy'/exp OR 'pregnant woman':ti,ab,kw OR 'pregnant woman'/exp OR newborn:ti,ab,kw OR 'newborn'/exp OR neonatal:ti,ab,kw OR neonatal OR gestation*:ti,ab,kw OR gestation* OR women:ti,ab,kw OR 'women'/exp OR mother:ti,ab,kw OR 'mother'/exp OR female:ti,ab,kw OR 'female'/exp OR 'pregnant mothers':ti,ab,kw OR 'pregnant mothers' OR 'antenatal mothers':ti,ab,kw OR 'antenatal mothers' OR 'antepartum women':ti,ab,kw OR 'antepartum women' OR neonate:ti,ab,kw OR 'neonate'/exp OR baby:ti,ab,kw OR 'baby'/exp OR child*:ti,ab,kw OR child* OR infant:ti,ab,kw OR 'infant'/exp OR infancy:ti,ab,kw OR 'infancy'/exp	
#2	risk:ti,ab,kw OR 'risk'/exp OR risks:ti,ab,kw OR risks OR probability:ti,ab,kw OR 'probability'/exp OR probabilities:ti,ab,kw OR probabilities OR causality:ti,ab,kw OR 'causality'/exp OR causation:ti,ab,kw OR causation OR 'enabling factor':ti,ab,kw OR 'enabling factor' OR 'enabling factors':ti,ab,kw OR 'enabling factors' OR 'predisposing factor':ti,ab,kw OR 'predisposing factor'/exp OR 'predisposing factors':ti,ab,kw OR 'predisposing factors' OR 'prognostic factors':ti,ab,kw OR 'prognostic factors' OR 'prognostic factor':ti,ab,kw OR 'prognostic factor'/exp OR 'prognostic determinants':ti,ab,kw OR 'prognostic determinants' OR determinants:ti,ab,kw OR 'determinants'/exp OR factors:ti,ab,kw OR factors OR factor:ti,ab,kw OR factor OR predictors:ti,ab,kw OR 'predictors'/exp OR 'predictor variable':ti,ab,kw OR 'predictor variable'/exp OR predictor:ti,ab,kw OR predictor	
#3	'prediction models':ti,ab,kw OR 'prediction models' OR 'prediction model':ti,ab,kw OR 'prediction model'/exp OR prediction:ti,ab,kw OR 'prediction'/exp OR predictive:ti,ab,kw OR predictive OR forecasting:ti,ab,kw OR 'forecasting'/exp OR 'prognostic model':ti,ab,kw OR 'prognostic model'/exp OR 'predictive model':ab,ti OR 'predictive model'/exp OR prognostic:ti,ab,kw OR prognostic OR predict*:ti,ab,kw OR predict*	
#4	stillbirth:ti,ab,kw OR 'stillbirth'/exp OR 'intrauterine fetal death':ti,ab,kw OR 'intrauterine fetal death'/exp OR 'fetus death':ti,ab,kw OR 'fetus death'/exp OR preterm:ti,ab,kw OR preterm OR prematurity:ti,ab,kw OR 'prematurity'/exp OR 'premature labor':ti,ab,kw OR 'premature labor'/exp OR 'preterm birth':ti,ab,kw OR 'preterm birth'/exp OR 'premature birth':ti,ab,kw OR 'premature birth'/exp OR 'low birth weight':ti,ab,kw OR 'low birth weight'/exp OR lbw:ti,ab,kw OR lbw OR 'small-for-gestational age':ti,ab,kw OR 'small-for-gestational age'/exp OR 'small for gestational age':ti,ab,kw OR 'small for gestational age'/exp OR 'small for date infant':ti,ab,kw OR 'small for date infant'/exp OR sga:ti,ab,kw OR sga OR 'fetal growth restriction':ti,ab,kw OR 'fetal growth restriction'/exp	
	#1 AND #2 AND #3 AND #4	21,288<br>February 24, 2022<br>3:08 PM
	Web of science	

#1	**Pregnant women** (Topic) or **Pregnant woman** (Topic) or **Pregnancy** (Topic) or **newborn** (Topic) or **neonatal** (Topic) or **gestation** (Topic) or **women** (Topic) or **mother** (Topic) or **female** (Topic) or **Pregnant mothers** (Topic) or **antenatal mothers** (Topic) or **antepartum women** (Topic) or **neonate** (Topic) or **baby** (Topic) or **child** (Topic) or **children** (Topic) or **infant** (Topic) or **infancy** (Topic)	
#2	**risk** (Topic) or **risks** (Topic) or **probability** (Topic) or **probabilities** (Topic) or **causality** (Topic) or **causation** (Topic) or **enabling factor** (Topic) or **enabling factors** (Topic) or **predisposing factor** (Topic) or **predisposing factors** (Topic) or **prognostic factors** (Topic) or **prognostic factor** (Topic) or **prognostic determinants** (Topic) or **determinants** (Topic) or **factors** (Topic) or **factor** (Topic) or **predictor** (Topic) or **predictors** (Topic)	
#3	**prediction models** (Topic) or **prediction model** (Topic) or **prediction** (Topic) or **predictive** (Topic) or **forecasting** (Topic) or **prognostic model*** (Topic) or **predictive model*** (Topic) or **prediction model*** (Topic) or **progn*** (Topic) or **predict*** (Topic)	
#4	**Premature Birth** (Topic) or **preterm labor** (Topic) or **Preterm delivery** (Topic) or **Prematurity** (Topic) or **premature labor** (Topic) or **low birth weight** (Topic) or **"low birthweight"** (Topic) or **"low birth weight"** (Topic) or **LBW** (Topic) or **"small-for-gestational age"** (Topic) or **"small for gestational age"** (Topic) or **SGA** (Topic) or **small for gestational age** (Topic) or **"fetal growth restriction"** (Topic) or **fetal growth restriction** (Topic)	
	**#1 AND #2 AND #3 AND #4**<br><br>**Filter eg. Language**	21,705<br>February 24, 2022<br>3:03 PM
	CINAHL	
S1	TI pregnant women OR AB pregnant women OR TI pregnancy OR AB Pregnancy OR TI newborn OR AB newborn OR TI neonatal OR AB neonatal OR TI gestation OR AB gestation OR TI women OR AB women	
S2	TI mother OR AB mother OR TI female OR AB female OR TI Pregnant mothers OR AB Pregnant mothers OR TI antenatal mothers OR AB antenatal mothers OR TI antepartum women OR AB antepartum women OR TI neonate OR AB neonate	
S3	TI baby OR AB baby OR TI child OR AB child OR AB children OR AB children OR TI infant OR AB infant OR TI infancy OR AB infancy	
S1+S2+S3=<br>S4<br>#1	S1 OR S2 OR S3<br><br>TI pregnant women OR AB pregnant women OR TI pregnancy OR AB Pregnancy OR TI newborn OR AB newborn OR TI neonatal OR AB neonatal OR TI gestation OR AB gestation OR TI women OR AB women OR TI mother OR AB mother OR TI female OR AB female OR TI Pregnant mothers OR AB Pregnant mothers OR TI antenatal mothers OR AB antenatal mothers OR TI antepartum women OR AB antepartum women OR TI neonate OR AB neonate OR TI baby OR AB baby OR TI child OR AB child OR AB children OR AB children OR TI infant OR AB infant OR TI infancy OR AB infancy	

S5	TI risk OR AB risk OR TI risks OR AB risks OR TI probability OR AB probability OR TI probabilities OR AB probabilities OR TI causality OR AB causality OR TI causation OR AB causation	
S6	TI enabling factors OR AB enabling factors OR TI predisposing factors OR AB predisposing factor OR TI prognostic factors OR AB prognostic factors OR TI prognostic determinants OR AB prognostic determinants OR TI determinants OR AB determinants OR TI risk factors OR AB factors	
S7	TI predictors OR AB predictors	
S8<br>#2	S5 OR S6 OR S7<br>TI risk OR AB risk OR TI risks OR AB risks OR TI probability OR AB probability OR TI probabilities OR AB probabilities OR TI causality OR AB causality OR TI causation OR AB causation OR TI enabling factors OR AB enabling factors OR TI predisposing factors OR AB predisposing factors OR TI prognostic factors OR AB prognostic factors OR TI prognostic determinants OR AB prognostic determinants OR TI determinants OR AB determinants OR TI risk factors OR AB factors OR TI predictors OR AB predictors	
S9	TI prediction model OR AB prediction model OR TI (prediction or predictive or predictors) OR TI (prediction or predictive or predictors) OR TI forecasting OR AB forecasting OR TI predictive model OR AB predictive model OR TI predictive modeling OR AB predictive modeling OR TI prediction model OR AB prediction model	
S10	TI prognostic factors OR AB prognostic OR TI prognostication OR AB prognostication OR TI (prognosis or predictor) OR AB (prognosis or predictor) OR TI prognosis prediction OR AB prognosis prediction OR TI (prognosis or outcome or survival) OR AB (prognosis or outcome or survival) OR TI (predict or predictors or predictive or prediction) OR AB (predict or predictors or predictive or prediction)	
S11<br>#3	S9 OR S10<br>TI prediction model OR AB prediction model OR TI (prediction or predictive or predictors) OR TI (prediction or predictive or predictors) OR TI forecasting OR AB forecasting OR TI predictive model OR AB predictive model OR TI predictive modeling OR AB predictive modeling OR TI prediction model OR AB prediction model OR TI prognostic factors OR AB prognostic OR TI prognostication OR AB prognostication OR TI (prognosis or predictor) OR AB (prognosis or predictor) OR TI prognosis prediction OR AB prognosis prediction OR TI (prognosis or outcome or survival) OR AB (prognosis or outcome or survival) OR TI (predict or predictors or predictive or prediction) OR AB (predict or predictors or predictive or prediction)	
S12	TI (stillbirth or still birth or intrauterine death or perinatal death or fetus death or foetus death) OR AB (stillbirth or still birth or intrauterine death or perinatal death or fetus death or foetus death) OR TI (preterm birth or premature birth or preterm labor or preterm delivery or prematurity) OR AB (preterm birth or premature birth or preterm labor or preterm delivery or prematurity) OR TI preterm labor OR AB preterm labor OR TI preterm delivery OR AB preterm delivery OR TI low birth weight OR AB low birth weight OR TI LBW OR AB LBW	
S13	TI small-for-gestational age OR AB small-for-gestational age OR TI small for gestational age OR AB small for gestational age OR TI SGA OR AB SGA OR TI fetal growth restriction OR AB fetal growth restriction	

S14<br>#4	S12 OR S13<br><br>TI (stillbirth or still birth or intrauterine death or perinatal death or fetus death or foetus death) OR AB (stillbirth or still birth or intrauterine death or perinatal death or fetus death or foetus death) OR TI (preterm birth or premature birth or preterm labor or preterm delivery or prematurity) OR AB (preterm birth or premature birth or preterm labor or preterm delivery or prematurity) OR TI preterm labor OR AB preterm labor OR TI preterm delivery OR AB preterm delivery OR TI low birth weight OR AB low birth weight OR TI LBW OR AB LBW OR TI small-for-gestational age OR AB small-for-gestational age OR TI small for gestational age OR AB small for gestational age OR TI SGA OR AB SGA OR TI fetal growth restriction OR AB fetal growth restriction	
S15	S4 AND S8 AND S11 AND S14<br><br>Filter by English	13,000<br>Wednesday, February 25, 2022 11:05 PM
	Google scholar	
	Prognostic prediction models for adverse birth outcomes, low birth weight, preterm, still birth	1017<br>February 25, 2022<br>Time 10:05 AM
	Others (WorldCat, AJOL, Hinary, Registery)	342<br>February 25, 2022<br>Time 11:00 AM
	Total: 60,194<br>• Data bases =58,835<br>• Registers (google scholar + others) =1359	
	Duplication removal<br>• Duplication records=15 184<br>• Screening articles by TAB (ongoing)<br>• Searching and appraisal of articles for SR (Preparing checklist	

Table S2: Study characteristics prediction models for low birth weight

Study	Country	Study Design	Center	Study Population	Outcome	Sample Size	Outcome(N)	Incidence	Events Per Variable	Number of predictors	Model Type	Model Analysis

						Develop ment	Validat ion						
Hange et al., 2018 (1)	USA	Cross-Sectional	Population Based	Women Neonates	Low Birth Weight	10,000	Not reporte d	Not reported	Not reported	Not reported	Gestational age, residence, sex, race of child, Education, Number of babies dead, Termination, Parity, Smoking, Weight Gained, Other medical conditions, Induction of labor, Cesarean section and BMI	Derivation	Machine Learning
Hassen et al., 2020 (2)	Ethiopia	Prospectiv e Cohort	Population Based	Pregnant Women	Low Birth Weight	379	Not reporte d	83	21.9%	13.8	Age at pregnancy, Underweight, Anemia, Height, Gravidity, Presence of comorbidity	Derivation	Logistic Regression
Herna¡n dez Castro et al., 2021 (3)	Mexico	Prospectiv e Cohort	Single Centered	All Pregnant Between 24 And 28 Weeks Of Gestation	Low Birth Weight	339	Not reporte d	19	5.6%	9.5	FG < 10th percentile, Null- Parity	Derivation Incremental Value	Logistic Regression
Goyal et,al, 2015a (4)	Greece	Retrospec tive Cohort	Multi-Centered	3rd Trimester Pregnancy	Low Birth Weight	263	146	30	11.4%	4.28	Multi-parous, Race, rape, Primi-Parous, BMI, Prenatal Care Entry > 28 Weeks, Prior Preterm Birth	Derivation	Logistic Regression
Goyal et,al, 2015b (5)	USA	Retrospec tive Cohort	Multi Centered	All Pregnant Women	Low Birth Weight	263	146	30	11.4%	4.28	Multi-parous, Race, Rape, Other Medical Conditions, Primi-Parous, Prenatal Care Entry > 28 Weeks, Prior Preterm Birth	Derivation	Logistic Regression
O'Reilly -Green and Divon ,1999 (6)	New York	Prospectiv e Cohort	Single Centered	Prolonged Pregnant Women	Low Birth Weight	410	Not reporte d	Not reported	Not reported	Not reported	Fetal Weight	Derivation	Logistic Regression
Griffin et,al, 2018 (7)	UK And Ireland	Prospectiv e Cohort	Multi Centered	2nd And 3rd Trimester Pregnant	Low Birth Weight	274	123	96	35%	9.6	Nephrin, CPA-4a , Sflt-1, Endoglin, PlGF , PlGF S-Flt Ratio , PlGF/Endoglin Ratio , PlGF + CPA-4a PlGF + Nephrin, PlGF + Nephrin + CPA-4a	Derivation	Logistic Regression
Ryu et al . , 2019 (8)	South Korea	Prospectiv e Observati onal	Single Centered	Pregnant Women With Singleton Pregnancies ,Who Is Normotensive And Preeclampsia	Low Birth Weight	140	140	140	40	Not reported	Serum UA, Total Bilirubin , Hemoglobin	Derivation	Logistic Regression
Schneue r et al. , 2014 (9)	Australia	Prospectiv e Cohort	Single Centered	Pregnant Women Attending First Trimester	Low birth weight	4621	Not reporte d	Not reported	Not reported	Not reported	Angiopoietin 1 ,Angiopoietin ,Angiopoietin 1/Angiopoietin 2 Ratio Maternal Age, socio-Economic status, Country Of Birth	Derivation	Logistic Regression
Bartnic	German	Experime	Single	Pregnant	Low Birth	112	Not	14	11.9%	Not	Fetal Fibronectin	Derivation	Logistic

ki et al, 1996 (10)	y	ntal	Centered	Women Between 22 And 35 Weeks	Weight		reporte d			reported			Regression
Cohen,et al 2014 (11)	USA	Retrospec tive, Case Control	Single Centered	Women Delivered of A Singleton Gestation	Low Birth Weight	2199		106	5%	Not reported	Pregnancy Associated Plasma Protein A (PAPP-A), Free BHCG, Maternal Serum Alpha Fetoprotein	Derivation	Logistic Regression
Sinding et al. , 2017 (12)	Denmar k	Prospectiv e Observati onal	Single Centered	Singleton Pregnancies	Low Birth Weight	97	Not reporte d	15	15%	Not reported	Placental T2, uterine Artery Pulsatility Index (UAPI)	Derivation	Logistic Regression
Singh et al. ,2014 (13)	India	Case Control	Single Centered	LBW Neonates and Normal Birth Weight Infants	Low Birth Weight	400	100	250	50%	3.3	Weight Gain, Inadequate Proteins, Previous PTB, Previous LBW Baby, Anemia and Smoking	Derivation	Logistic Regression
Wu et al. , 2021 (14)	China	Cross Sectional	Multi Centered	Singleton Live Infants and Their Mothers	Low Birth Weight	27233	Not reporte d	1667	6.1%	Not reported	Sex, Gestational Age, Education ANC, Occupation, Pregnancy Induced Hypertension, Income, Pesticide, Nutrients	Derivation	Logistic Regression
Zahirzad a and lavagnan anda ,2021 (15)	Afghanis tan	Cross Sectional	Single Centered	Both Low Birth Weight and Normal Weight Babies	Low Birth Weight	2120	Not reporte d	625	29.5%	Not reported	Residence	Derivation	Logistic Regression
Reeb et al. ,1987 (16)	USA	Cross Sectional	Single centered	Pregnant women in their seventh month of gestation	Low birth weight	140	Not reporte d	20	14%	Not reported	Prenatal stressful events, Maternal social support, Maternal depression, BMI, Family functioning, bio medical and psychosocial factors	Derivation	Logistic regression
Rezaei et al. ,2016 (17)	Iran	Cross-sectional	Single centered	Women and alive born infants	Low birth weight	245	Not reporte d	Not reported	Not reported	Not reported	Gestational age, rank of birth, type of delivery, sex	Derivation	Logistic regression

Table S2: Study characteristics prediction models for low birth weight (continued)

Study	Model Development	Model Evaluation	Internal Validation	External Validation	Calibration	Model Discrimination (AUC With 95% CI)	Model Performances	Sensitivity With 95%CI	Specificity With 95%CI	Handling Of Mission Value	Method Of Handling Missing Value	Model Selection	Handling Of Continuous Data	How Is The Model Presented
Hange et al., 2018 (1)	By Using Registry Data Model Was Developed Before Balancing And After Balancing. The Study Uses Correlation-Based Feature Selection Algorithm (CFS) Model Was Built With J48 Decision Tree, REPtree And Random Tree.	-AUC<br>-Sensitivity<br>-Specificity	Not reported	Not reported	No	-Before J48=0.599(NR, NR)<br>Reduced-error pruning tree(REPtree)=0.629(NR,NR)<br>-Random Tree=0.518(NR, NR)<br>-After J48=0.903(NR, NR)<br>REPtree=0.884(NR, NR)<br>-Random Tree=0.853(NR, NR)	Yes	-Before Data Balancing J48=36.8%(NR,NR)<br>REP Tree=39.2 % (NR, NR)<br>Random Tree= 28.6%(NR,NR)<br>After Balancing J48=66.3% (NR, NR)<br>REPtree=61.7%(NR,NR)<br>Random Tree=65 % (NR, NR)	Before Data Balancing J48=74.9%(NR, NR)<br>REPtree=73.4% (NR, NR)<br>Random Tree=74.7% (NR, NR)<br>-After Data Balancing J48=95.4% (NR, NR)<br>REPtree=94.9% (NR, NR)<br>Random Tree=95.5% (NR, NR)	Yes	Single Regression Imputation	Correlation-Based Feature Selection	Kept Linear	Not reported
Hassen et al., 2020 (2)	The Model Developed Using Prospective Cohort Pregnant Women. Develop A Simplified Risk Score and Evaluated By AUC And Calibration Plot. Use Bootstrapping Technique And A Decision Curve Analysis Was Done	-AUC<br>Calibration Plot<br>Sensitivity<br>Specificity<br>PPV<br>NPV	Bootstrapping	No	P-Value; 0.98	0.83(0.779, 0.879)	Yes	73 % (60%, 81%)	82 % (77%, 86%)	Yes	Multiple Imputation	Stepwise Backward Selection	Categorized	Risk Score
Herna¡ndez Castro et al., 2021 (3)	The Model Was Developed By Doing Multivariate Logistic Regression Analysis Used Goodness-Of-Fit Using X2 Wald,	AUC<br>Sensitivity<br>Specificity<br>NPV<br>PPV	Not reported	Not reported	Not reported	-Null Parity =0.60(0.55,0.65)<br>-FG < 10th= 0.69(0.59,0.70.<br>-Combined =0.72(0.67,0.77)	Yes	For Null-Parity =67.61% For FG < 10th = 44.13% For Combined=23.53%	For Null-Parity=52.15% For FG < 10th =85.29%<br>For Combined =93.44%	Not reported	N/A	Not reported	Categorized	Prediction Rule

	Their Predictive Capacity Was Determined By Area Under The Curve (AUC) Analysis.													
Goyal et,al, 2015a (4)	Not reported	AUC	Splitting	No	No	0.63(NR, NR)	Yes	29%	80%	Not reported	N/A	Stepwise Selection (Forward Selection Followed By Series Of Backward Selection)	Categorized	Risk Score Chart
Goyal et,al, 2015b (5)	Use Stepwise Elimination Of Variable To Develop Model	AUC	Splitting	No	Not reported	0.63(NR, NR)	Yes	29%	82%	Yes	Complete Case Analysis Multiple Imputation	Stepwise Selection (Forward Selection Followed By Series Of Backward Selection)	Categorized	Risk Score
O'Reilly-Green and Divon ,1999 (6)	Model Develop By Collecting Data From Neonates And Evaluated	AUC Sensitivity Specificity	Not reported	No	Not reported	0.961(NR, NR)	Yes	94.1%	88.30%	Not reported	N/A	Not reported	Kept Linear	Regression Coefficients
Griffin et,al, 2018 (7)	To Develop The Model Use Stepwise Logistic Regression For Bio Markers	AUC, Sensitivity, Specificity	Splitting	No	Not reported	0.83(NR, NR)	Yes	89.7% (91.7%,94.7%)	58.7% (51.1%,66%)	Yes	Complete Case Analysis	Stepwise Selection (Forward Selection Followed By Series Of Backward Selection)	Kept Linear	Prediction Rule
Ryu et al . , 2019 (8)	The Model Was Developed from Preeclampsia And Normal Pregnant Women With Uric Acid As A Predictor	AUC Sensitivity Specificity	Bootstrapping	No	Not reported	0.902 (0.817, 0.986)	Yes	58%	95%	No	N/A	Not reported	Kept Linear	Model Formula with Regression Coefficients
Schneuer et al. , 2014 (9)	The Model Was Developed From Maternal Characteristics Like Age, Weight, Country Of Birth, Socio Economic Status With The	AUC Sensitivity Specificity	Not reported	Not reported	Not reported	Ang-1/Ang-2 Ratio; 0.70 (NR, NR) Maternal Risk Factors 0.58(NR, NR)	Yes	<10%	Not reported	Yes	Multiple Imputation	Not reported	Not reported	Algorism

	Above Predictors													
Bartnicki et al, 1996 (10)	The Model Develops By Fibronectin To Predict LBW	Sensitivity Specificity	Not reported	Not reported	Not reported	Not reported	Yes	85.7%	75.9%	No	N/A	Stepwise Selection (Forward Selection Followed by Series of Backward Selection)	Kept Linear	Regression Coefficients
Cohen,et al 2014 (11)	The Model Develops From Pregnancy Associated Proteins	AUC Sensitivity, Specificity	Not reported	Not reported	Not reported	0.70(NR.NR)	Yes	69%	63%	Yes	Complete Case Analysis	Univariable And Multivariable Logistic Regression	Kept Linear	Regression Coefficients
Sinding et al. , 2017 (12)	The Model Was Developed By Placental T2* And The Uterine Artery (Uta) Pulsatility Index (PI) As Predictors Of Low Birth Weight Measured By Gradient Recalled Multi-Echo MRI Sequence And Doppler Ultrasound Respectively	AUC Sensitivity Specificity	Not reported	Not reported	Not reported	-Placenta T2= 0.92(0.85,0.98)<br>Uta PI = 0.74(0.60,0.89)	Yes	Placenta T2= 93 % (68%, 100%)<br>Uta PI Z-Score 60 % (32%, 84%)	Placenta T2; 87% (77%, 93%) Uta=83 % (73%, 90%)	No	N/A	Not reported	Kept Linear	Prediction Rile
Singh et al. ,2014 (13)	The Model Was Developed By Splitting Samples For Development And Validation 400 And 100 Respectively Starting From Cases Versus Control Ask Back To The Exposure	AUC Sensitivity Specificity	Splitting	Not reported	Not reported	0.79(NR.NR)	Yes	At Cut-Off Score 29.25 = 71.6 %, For A Score Of 24.75 =73.7 %	At Cut-Off Score 29.25 = 67.0 %,.<br>For A Score Of24.75 =64 %,	No	N/A	Not reported	Kept Linear	Risk Score Chart
Wu et al. , 2021 (14)	The Model Was Developed By Comparing Trans Vaginal And Trans Perennial Sonographic To Predict Preterm Delivery By Examining At 24	AUC Sensitivity Specificity	Not reported	Not reported	Not reported	For Trans Vaginal Sonographic=0.801(NR, NR)<br>For Trans Perennial Sonographic= 0.857(NR, NR)	Yes	Trans Vaginal Sonographic=72%<br>Trans Perennial Sonographic= 77%	Trans Vaginal Sonographic=81.8% Trans Perennial Sonographic= 82.7%	No	N/A	Not reported	Kept Linear	Algorism

	Weeks Gestation													
Zahirzada and lavagnananda ,2021 (15)	The Model Was Developed By Using Afghanistan DHS Data To Predict LBW At Residence Classification Urban Vs Rural	AUC	Not reported	Not reported	Not reported	Rural=0.91(NR, NR) Urban=0.92(NR,NR)	No	Not reported	Not reported	Yes	Single Regression Imputation	Not reported	Categorized	Algorism
Reeb et al. ,1987 (16)	The model was developed from demographic, bio medical and psychosocial factors in urban black pregnant women	Sensitivity Specificity	Not reported	Not reported	Not reported	Not Reported	Yes	65%	84%	No	N/A	Stepwise forward selection	Categorized	Regression coefficients
Rezaei et al. ,2016 (17)	The model was developed from maternal and infant characteristics	AUC and sensitivity	Not reported	Not reported	Not reported	0.888(NR, NR)	Yes	85.50%	90.9%	No	N/A	Stepwise forward selection	Kept linear	regression coefficients

Table S3: Study characteristics prediction models for preterm birth

Study	Country	Study Design	Center	Study Population	Outcome	Sample Size		Outcome(N)	Incidence	Events Per Variable	Number Of Predictors	Model Type	Model Analysis
						Development	Validation						
Owen et al, 1990 (18)	USA	Prospective Follow Up	Single Center	Singleton Gestations Pregnant Women	Preterm Birth	7478	Not reported	1122	16.4 %	For PTB LRG=7.1 HRG=16.4 VLPTB LRG=1.6 HRG=4.7	Pre-Pregnancy Weight gain	Derivation	Logistic Regression
Carter,et al 2020 (19)	UK	Prospective Cohort	Multi-Center	2nd & 3rd Trimester Pregnant Women	Preterm Birth	1032	506	<30 Weeks = 22 <34 Weeks = 60 <37 Weeks = 144	12.3%	<30 Weeks= 4.4 <34 Weeks = 12 <37 Weeks = 28.8	Previous PTB/PPROM, Previous Late Miscarriage, Cervical Surgery, Twin Pregnancy	External Validation	Parametric Survival Model
Celik et,al, 2008 (20)	UK	Prospective Observational	Multi – Center	2nd Trimester Pregnancy	Preterm Birth	58807	Not reported	3237	0.99%	539.5	Race, BMI, Smoking, Previous preterm labor, Cervical Length	Incremental Value	Logistic Regression

Crane and Hutchens 2008 (21)	Canada	Retrospective Cohort	Single Center	2nd & 3rd Trimester Pregnancy	Preterm Birth	90	103	11	12.22%	2.2	Gravidity, Parity, Bleeding, Poly-Hydramnios	Impact Study	Logistic Regression
Damaso et,al, 2019 (22)	Brazil	Retrospective Cohort	Single Center	1st Trimester Pregnancy	Preterm Birth	1323	Not reported	110	8.31%	55	Smoking, Spontaneous PTB	External Validation	Logistic Regression
He et al., 2018 (23)	China	Prospective Cohort	Multi-Center	Pregnant Women Who Attended Their First ANC(16 Weeks)	Preterm Birth	9044	Not reported	444	4.9%	Not reported	Maternal Age, Maternal Height, History of Preterm Delivery, Vaginal Bleeding, Lack of Folic Acid intake	Derivation External Validation	Logistic Regression Proportional Hazard Model
Hebbar and samjhana 2006 (24)	India	Randomized Prospective Longitudinal Study	Single-Center	Pregnant Women At 20 - 24 Weeks Of Gestational Age	Preterm Birth	168	Not reported	13	7.7%	13	Cervical Length	Derivation	T-Test
Hebert et al., 2022 (25)	USA	Retrospective Cohort	Single Center	All Women Aged 15 To 44 Who Had A Live Birth	Preterm Birth	349,396	10,000	Development N= 6,330 Validation N= 186	1.86%	Not reported	Not reported	Derivation	Logistic Regression
Newman et al. (26)	USA	Prospective Cohort	Multi Center	Singleton Pregnancies	Preterm Birth	2916	Not reported	127	4.4%	Not reported	Maternal Age, BMI, Education, Insurance, Marital Status, Race, Parity, Previous Pre-Term Delivery, Smoking, Alcohol Drinking, Bishop Score	Derivation	Logistic Regression
Hellemans et al., 1995 (27)	Belgium	Prospective Follow Up	Single Center	Pregnant Women Singleton Gestation	Preterm Birth	133	Not reported	10	8%	8	Cervical Fetal Fibronectin	Derivation	Not reported
Fergus et,al, 2013 (28)	UK	Retrospective Cohort	Single Center	2nd & 3rd Trimester Pregnant Women	Preterm Birth	240	60	38	12.67%	38	Electro-Hysterography (EHG)	Derivation	Machine Learning
Jelliffe-Pawlowski et al., 2018 (29)	USA	Nested Case Control	Population Based	All Women With Singleton Births	Preterm Birth	200	200	200	50%	7.69	Gestation Age, Income and Biomarkers	Derivation	Logistic Regression
Goyal et,al, 2015a	Greece	Retrospective Cohort	Multi-Center	3rd Trimester Pregnancy	Preterm Birth	263	146	22	8.4%	3.14	Parity, Race, Rape, BMI, Prenatal Care, Prior Preterm Birth	Derivation	Logistic Regression

(4)													
Jung et al., 2021 (30)	Korea	Prospective Cohort	Multi Center	Women With Singleton Pregnancies 16 - 28 Weeks Of Gestation	Preterm Birth	115	Not reported	9	0.69%	2.25	Mean Internal Os Strain Mean External Os Strain Elasticity Contrast Index	Derivation	Logistic Regression
Goyal et,al, 2015b (5)	USA	Retrospective Cohort	Multi Centered	All Pregnant Women	Preterm Birth	263	146	22	8.4%	3.14	parity, Race, Rape, Other Medical Conditions, Prenatal Care Entry, Prior Preterm Birth	Derivation	Logistic Regression
Nikolova , et al.2018 (31)	Finland , Macedonia And Russia	Prospective Observational	Multi Centered	Pregnant Women Singleton Gestations	Preterm Birth	Not reported	383	65	16.9%	Not reported	PAMG-1,Phigfbp-1 and Cervical Length	Incremental Value	Logistic Regression
Alleman, et al 2013 (32)	USA	Prospective Cohort	Not reported	All Women Undergoing Routine Perinatal Testing in The First Second Trimester	Preterm Birth	2699	Not reported	200	0.074	Not reported	TC, Pre-Pregnancy DM, Previous PTB, Previous Live Birth, BMI, maternal AFP, maternal Inhibin A	Derivation	Logistic Regression
Anderson, et al 1990 (33)	USA	Prospective Follow-Up	Not reported	Women First Seen for Routine Obstetric Ultrasonography Examination	Preterm Birth	178	Not reported	44	24.7%	Not reported	-Trans Abdominal Ultrasonography, Endo-vaginal Ultrasonography and Manual Examination Cervical Length	Derivation	Logistic Regression
Grobman et,al, 2016 (34)	USA	Clinical Trial	Single Centered	2nd Trimester Pregnant	Preterm Birth	657	Not reported	109	16.6%	54.5	Mean Cervical Length, Presence of Cervical Funneling	Impact Study	Logistic Regression
Park et al., 2021 (35)	Korea	Case Control	Multi-Centered	Singleton Pregnant Women with A Gestational Age Between 15 And 34 Weeks	Preterm Birth	94	Not reported	38	40.4%	Not reported	Bacillus Bacteroidetes, Molicutes and WBC	Derivation	Logistic Regression
Pasquini et al.,2014 (36)	Italy	Retrospective Observational	Single Centered	Asymptomatic Twin Pregnancies Who Received ANC Ultrasound Examination At 20 And 24 + 6 Weeks	Preterm Birth	222	Not reported	44	19.8%	Not reported	Cervical Length	Derivation	Logistic Regression
Posthumus et al., 2016 (37)	Netherland	Prospective Cohort	Multi Centered	All Pregnant Women	Preterm Birth	836	Not reported	101	12%	Not reported	Previous History of PTB, Smoking, Alcohol Drinking, Drug Abuse, Social Support, Family Income, Housing	Derivation	Logistic Regression

											Condition		
Dziadosz et,al, 2016 (38)	New York	Retrospective Cohort Study	Single Centered	2nd Trimester	Preterm Birth	972	Not reported	<37 Weeks =84 <34 Weeks = 43	9.6%	<37 Weeks=8.4 <34 Weeks = 4.3	Prior Spontaneous PTB, Prior Dilation and Curettage , Gestational Age ,Mean CL ,Mean UCA at TVU ,BMI, Cesarean Delivery , NICU Admission	Not reported	Linear Regression
Prema and pushpalatha ,2019 (39)	India	Prospective Observational Study	Single Centered	Pregnant Women Having Who Had DM	Preterm Birth	Not reported	Not reported	124	Not reported	Not reported	Maternal Age, Gravidity, Diabetes, Obesity, Hypertension	Derivation	Logistic Regression
Garcia – blanco et,al, 2017(40)	Spain	Prospective Cohort	Single Centered	2nd And 3rd Trimester	Preterm Birth	166	Not reported	128	77	14.2	Parity, Cortisol With Or Without Amylase, maternal Age, Multiple Pregnancy	Derivation	Parametric Survival Model
Raba and Kotarski ., 2016 (41)	Poland	Prospective Observational	Single Centered	Pregnant Women	Preterm Birth	622	Not reported	400	64.3%	Not reported	Smoking, Socioeconomic Status, Frequent Contractions During Pregnancy, vaginal Bleeding, other medical condition	Derivation	Logistic Regression
Rafael et al. , 2012 (42)	USA	Retrospective Cohort	Single Centered	Women With A Twin Delivery Who Subsequently Had A Singleton Delivery	Preterm Birth	255	Not reported	18	7.05%	Not reported	Gestational Age	Derivation	Logistic Regression
Crane et,al, 1999 (43)	Canada	Prospective Cohort	Single Centered	2nd Trimester Pregnant	Preterm Birth	140	Not reported	9	6.4%	4.5	Preterm Birth-Risk Score, Vaginal Fetal Fibronection ,FDC-6	Impact Study	Logistic Regression
Baer et al 2018 (44)	USA	Retrospective Cohort	Birth Cohort Database	First Trimester Pregnant	Preterm Birth	1,559,797	779,899	Training = 102, 947 Testing = 51,473	6.6%	Not reported	Race, Age, Education, Parity, Country of birth, BMI, Diabetes, Hypertension, smoking, Drug Abuse, Mental Illness, Anemia, Previous Cesarean Delivery, Previous PTB, Interpregnancy Interval	Derivation	Logistic Regression
Riboni et al. ,2011 (45)	Italy	Prospective Cohort	Multi Centered	Women A Singleton Pregnancy At 24-34 Weeks	Preterm Birth	210	Not reported	36	16.2%	Not reported	Phosphorylated Insulin-Like Growth Factor, Bindingprotein-1 Fetal Fibronectin	Derivation	Logistic Regression
Banos et al, 2018	USA	Prospective Cohort	Single Centere	All Women Attending	Preterm Birth	749	Not reported	29	5.45%	Not reported	Maternal Age, Cervical Length, Cervical	Derivation	Logistic Regressio

(46)			d	Second-Trimester At 19-And 24-Weeks Gestation							Consistency Index, Onset of Labor, Mode of Delivery		n
Riboni et al. ,2012 (47)	Italy	Prospective Cohort	Single Centered	Asymptomatic Pregnant Women 24 Weeks	Preterm Birth	491	Not reported	44	8.9%	Not reported	Phosphorylated Insulin-Like Growth Factor, Binding Protein (Phigfbp-1) Test, Cervico-Vaginal Interleukins 6 (IL-6) and 8 (IL-8), Serum C-Reactive Protein	Derivation	Logistic Regression
Kim et al., 2011 (48)	Korea	Retrospective Chart Review	Single Centered	Patients Diagnosed With Preterm Labour	Preterm Birth	175	Not reported	102	58.29%	11.33	WBC, CRP, NLR, Cervix Length and Combined, marker (Cervical Length and NLR)	Derivation	Proportional Hazard Model
Balardi, et al 2016 (49)	Italy	Exploratory Study	Multi Centered	Mothers Who Had Undergone An Amniocentesis Between 21 And 28 Gestational Weeks	Preterm Birth	32	Not reported	13	40.6%	Not reported	Gestational age, Sex, Birth Weight, Cesarean Section, Maternal Age, BMI, Smoking, Ethnicity, Previous Miscarriages, Trans-Abdominal Amniocentesis, Maternal Therapy at Amniocentesis	Derivation	Discriminant Analysis
Ridout et al., 2019 (50)	London	Retrospective Cohort	Multi Centered	Pregnant Women With Asymptomatic High-Risk Women With Congenital Uterine Anomaly	Preterm Birth	318	Not reported	56	18	Not reported	Cervical Length<br>Fetal Fibronectin	Incremental	Not reported
Gioan et,al, 2018 (51)	France	Prospective Cohort	Multi Centered	All Pregnant Women	Preterm Birth	813	Not reported	24	10.9%	4.8	History of PTB/PROM, Gestational Age, Cervical Length, Day Travel Time > 30 Minutes	Incremental Value	Logistic Regression
Koivu and sairanen, 2020 (52)	USA	Cross Sectional	Population Based	Infant Birth And Death Data Sets Containing Pregnancies During The Years CDC, National Center Of Health Statistics	Preterm Birth	15,883,784	363,560	1,532,538	9.6%	Not Stated	Maternal Age, Race, Marital Status, Education, previous PTB, Special Supplemental Nutrition Program, Smoking, BMI, Height, Weight, Parity, Pre-Pregnancy Diabetes, Pre-Pregnancy Hypertension, Infertility Treatment, , Assisted Reproductive Technology, Previous Cesarean Sections, other medical condition	Derivation	Logistic Regression
Barinov et al ,	Russia	Retrospective	Single Centere	Women With Singleton And	Preterm Birth	420	Not reported	420	100%	Not reported	Phosphorylated Insulin-Like Growth Factor	Incremental Value	Logistic Regressio

2021 (53)		Cohort And Prospective Observational	d	Twin Pregnancies Between 24 And 33 + 6 Weeks Of Gestation							Binding Protein-1 (Phigfb-1), Placental Alpha Macroglobulin-1(PAMG-1)		n
Kuhrt et al, 2016 (54)	UK	Prospective Follow up	Multi Centered	Women With A Singleton Pregnancy 22+0 And 35+6weeks	Preterm Birth	190	192	49	13%	24.5	Fetal Fibronectin and Previous PTB/PPROM	Derivation	Parametric Survival Model
Rohlfing et al. ,2020 (55)	USA	Case Control	Single Centered	All Pregnant Women Singleton Birth	Preterm Birth	868	Not reported	411	47.35%	Not reported	Hypertension, Diabetes Mellitus, Total Cholesterol(HDL,LDL,TC, TG),Smoking "	Derivation	Logistic Regression
Bartnicki et al, 1996 (10)	Germany	Experimental	Single Centered	Pregnant Women Gestational Age Between 22 Weeks And 35 Weeks,	Preterm Birth	112	Not reported	40	35.7%	Not reported	Fetal Fibronectin	Derivation	Logistic Regression
Bastek et al , 2012 (56)	USA	Prospective Cohort	Single Centered	Singleton Pregnancies At 22-33 Weeks Of Gestation	Preterm Birth	583	Not reported	Delivery Within 10 Days =90 , Before 37 Weeks = 204	35.0%	Not reported	Cervical Dilatation ,Prenatal Care, smoking and Obstetric History	Derivation	Logistic Regression
Saade et al. , 2016 (57)	USA	Nested Case Control Cohort	Multi Centered	Pregnant Women Between 17 And 28 Weeks Gestational Weeks	Preterm Birth	N/A	4825	533	11.04%	Not reported	-Insulin-Like Growth Factor-Binding Protein 4 (IBP4) ,Sex Hormone Binding Globulin (SHBG)	External Validation	Logistic Regression
Lee et al., 2011 (58)	Korea	Prospective cohort	Single centerd	women who delivered singleton live newborns from 24 to 42 weeks of gestation at Ewha Womans University Hospital in Seoul, Korea, between 2003 and 2010	Preterm birth	522	Not reported	96	18.4%	9.6	Maternal age, BMI, Gestational age , parity, Prior preterm birth, Maternal education, Paternal education, cervix length , fetal fibronectin	Derivation	Logistic regression
Arabi Belaghi et al,	Canada	Population-Based Retrospec	Single Centered	All Nulliparous Women With Singleton Births	Preterm Birth	75,809	37,654	6,955	6.2%	Not reported	Diabetes, Abnormal Pregnancy-Associated Plasma Protein A	Derivation	Logistic Regression

2021 (59)		tive Cohort		Who Gave Birth Between 20 And 42 Weeks Gestation							,Pregnancies Conceived By IVF, Being Obese, drug Use, Lower Neighborhood Education, Neighborhood Immigration , Maternal Height, Other health Conditions., obstetric history, Sex, Prenatal care		
Schmitz et al. , 2008 (60)	France	Prospective Cohort	Single Centered	Pregnant Women In Preterm Labor Between 24 And 34 Weeks Gestation.	Preterm Birth	395	Not reported	Within 48 Hour 17 and 7 days 32	8.1% ,	Not reported	Bishop Score Cervical Length	Derivation	Logistic Regression
Lee et al., 2020 (61)	Korea	Retrospective Cohort	Multi Centered	Pregnant Women Between 16 And 34 Gestational Weeks	Preterm Birth	727		452	62.2%	53.3	Pelvic Fall Down Sensation, Regular Physical Activity, Feeling of Uterine Contraction, Maternal Weight, History of Cerclage, Vaginal Bleeding, C- Reactive Proteins Level, other medical condition, Sleep Quality, WBC, Alcohol Drinking, Gestational Age, Rupture of Amniotic Membrane, Multiple Pregnancy	Derivation	Logistic Regression
Menon et al ,2014 (62)	USA	Case Control	Single Centered	Pregnant Women Between The Ages Of 18 And 40 Years	Preterm Birth	Not reported	Not reported	Not reported	Not reported	Not reported	In African Americans<br>- Maternal Plasma Samples IL-1RA, TNF-A, Angiopoietin 2, TNFRI, IL-5, MIP1a, IL-1b and, TGF-A<br>In Caucasians<br>- TNFR1, ICAM-1 and IL-1RA	Derivation	Multivariate Adaptive Regression Splines
Lee et al., 2015 (63)	Korea	Retrospective Cohort	Not reported	Women With A Viable Twin Pregnancy At 15 To 20 Weeks Of Gestation	Preterm Birth	192	Not reported	34	17.70%	17	Amniotic Fluid IL-8 and MMP-9	Derivation	Not reported
Leow et al., 2020 (64)	Australia	Prospective Cohort	Multi Centered	Pregnant Women Attending ANC Asymptomatic Women Of At Least 18 Years Of Age And 16-24 Weeks	Preterm Birth	136	150	Development 12 and validation=11	8.04%	3.29 (1.71 For Development And 1.57 For Validation)	IL-1RA, VDBP, TIMP-1, PEDF, GGH, LAMC2, ECM1	Derivation	Not reported

				Gestational Age									
Cohen et al ,2014 (11)	USA	Retrospective, Case Control	Single Centered	Women Delivered Of A Singleton Gestation	Preterm Birth	2199	Not reported	150	7%	Not reported	Pregnancy Associated Plasma Protein A (PAPP-A), Free BHCG and Maternal Serum Alpha Fetoprotein	Derivation	Logistic Regression
Leung et al., 2005 (65)	China	Prospective Observational	Single Centered	Women Attending This Routine Ultrasound Session	Preterm Birth	2880	Not reported	200	6.90%	1000	Cervical Length and Funneling	Derivation	Logistic Regression
Lin et al., 2021 (66)	USA And Columbia	Not reported	Single Centered	ART Women From The National Vital Statistics System (NVSS) Participant Use File.	Preterm Birth	122430	Not reported	15574	12.72%	1198	Age, Race, BMI, Parity, History of PTB and Cesarean, Birth Interval, Pre-pregnancy Diabetes, Pre-pregnancy Hypertension and Fertility-Enhancing Drugs	Derivation	Proportional Hazard Model
Liu et al., 2020 (67)	China	Retrospective Cohort	Single Centered	All Women Had Their Delivery At Peking University First Hospital In Beijing, China, From 1 January 2017 To 1 January 2018 With Short Cervix	Preterm Birth	555	Not reported	187	33.7%	20.78	BMI, other medical condition, History Of PTB, Twin Pregnancy, IVF-ET, inter pregnancy interval, Gestational Age, Cervical Length and Pre-pregnancy Hypertension	Derivation	Logistic Regression
Van de Mheen et al . ,2014 (68)	Netherland	RCT	Single Centered	Pregnant Women For AMPHA Drug Trial	Preterm Birth	507	Not reported	270	53%	Not reported	Cervical Length, Previous Preterm Delivery, Mono Chorionicity, Smoking, Educational Level and twin Pregnancy	Derivation	Proportional Hazard Model
Yazici et al. ,2004 (69)	Turkey	Prospective Observational	Single Centered	Pregnant Women Underwent Trans Vaginal Ultra Sound And Trans Perennial Ultra Sound At 24 Weeks Of Gestation	Preterm Birth	357	Not reported	22	6.2%	Not reported	Trans Vaginal Sonography and trans Perennial Sonography Measuring Cervical Length	Derivation	Logistic Regression
Mueller-Heubach	USA	Prospective Cohort	Single Centere	Patients Registering For	Preterm Birth	N/A	4591	462	10.1%	92.4	Pre-Pregnancy Weight gain , race, marital Status	External Validation	Logistic Regressio

Guzick., 1989 (70)			d	Prenatal Care									n
Mortensen et al.,1987 (71)	Sweden	Prospective Cohort	Not reported	Women Who Were Delivered	Preterm Birth	1327	Not reported	43	3.20%	21.5	Bishop Cervical Score and Dilatation	Derivation	Not reported
Morken et al., 2014 (72)	Sweden	Prospective Cohort	Population Based	Women Born Spontaneous Onset Of Delivery	Preterm Birth	448,852	430,139 (Primi-parous (190 936) And Multiparous (N239 203))	8569 In Primi-parous and 6489 In Multiparous	4.3%	Not reported	BMI, Smoking, Pre-Existing Diabetes, Hypertension, gestational age, sex, fetal complication, Pregnancy Bleeding, Previous Spontaneous Abortions.	Derivation	Logistic Regression
Zhang et al .,2021 (73)	China	Case Control	Single Centered	Twin Pregnant Women Between 22-24 And 26-28 Gestation	Preterm Birth	309	178	173	56%	Not reported	Parity, Monotonicity Pre Pregnancy's BMI Previous Preterm Birth, Cervical Length, Shortening Rate	Derivation	Logistic Regression
Macdonald – wallis et,al 2015 (74)	UK	Prospective Cohort	Multi Centered	3rd Trimester Pregnancy	Preterm Birth	12996	3005	702	5.4%	70	BMI, Height, Age, Parity, Smoking, previous existing Hypertension and Diabetes, Ethnicity	Derivation	Not reported
Mailath - pokorny et,al, 2015 (75)	Australia	Prospective Cohort	Single Centered	2nd Trimester Pregnancy	Preterm Birth	617	Not reported	300	48.6%	100	Preterm PROM, Cervical Length, CPR	Derivation; External Validation	Logistic Regression
McLean et,al, 1999 (76)	Australia	Prospective Cohort	Single Centered	1st And 2nd Trimester	Preterm Birth	860	Not reported	60	70	30%	Concentrations of Corticotrophin-Releasing Hormone, Fetoprotein	Derivation	Not reported
Watson et al. ,2020 (77)	UK	Prospective Cohort Study	Multi Centered	Asymptomatic Singleton Or Twin Pregnancies	Preterm Birth	1803	904	453	50.1%	Not reported	Quantitative Fetal Fibronectin, Cervical Length	Incremental Value	Proportional Hazard Model
Mehta-Lee et,al, 2017 (78)	USA	retrospective cohort study	Multi centered	postnatal women	Preterm birth	192,208	Not reported	47654	9.2%	5294.9	Obstetric history, History of preterm labor, SAB/TAB, maternal diabetes, Race, Ethnicity, smoking, stress over paying bills, intend to get pregnant, maternal age	Derivation	Logistic regression
Mendoz	Spain	prospectiv	Single	1st, 2nd and 3rd	Preterm birth	31	Not	14	45.2%	14	Cervical pessary placement	Derivation	paired

a et,al, 2021 (79)		e observational	centered	trimester pregnant women			reported						sample t-test
Zhu et al . ,2017 (80)	China	Prospective follow up	Multi centered	Pregnant women at 17 to 28+6 weeks	Preterm birth	130	Not reported	46	35.4	Not reported	History of preterm delivery, pre pregnancy BMI , blue area, , mean gray value(Amplitude), cervical dilatation, Cervical length	Derivation	Logistic regression
Radhouane et al. ,2017 (81)	Tunsia	Prospective longitudinal study	Single centered	Women with singleton pregnancies	Preterm birth	117	Not reported	40	34.2%	Not reported	Cervical length	Incremental value	Student t -test and Pearson chi-square test
Bachkangi et al ,2019 (82)	UK	prospective cohort study	Multi centered	pregnant women between 24 and 34 gestational week	Preterm birth	217	Not reported	71	32.7%	35.5	N-arachidonoylethanolamine (AEA) N-oleoylethanolamide (OEA)	Derivation	Logistic regression
Szymusik et al. ,2011 (83)	Poland	Prospective cohort	Single centered	Women in singleton pregnancies attending a second trimester scan at 22-24 weeks of gestation	Preterm birth	451	Not reported	100	22%	Not reported	Cervical length , Spontaneous singleton delivery and gestational age	Derivation	Logistic regression
Schaaf et al. ,2012 (84)	Netherland	Prospective cohort study	Population based	Singleton pregnancies	Preterm birth	1,524,058	1,524,058	57,796	3.80%	Not reported	- Previous PTB, Drug abuse, Vaginal bleeding, socio-economic status, parity	Derivation	Logistic regression

Table S3: Study characteristics prediction models for preterm birth (continued)

Study	Model Development	Model Evaluation	Internal Validation	External Validation	Calibration	Model Discrimination (AUC With 95% CI)	Model Performances	Sensitivity With 95%CI	Specificity With 95%CI	Handling Of Mission Value	Method Of Handling Missing Value	Model Selection	Handling Of Continuous Data	How Is The Model Presented

Owen et al, 1990 (18)	Not reported	Not reported	Not reported	No	Not reported	Not reported	Yes	Delivery <37 Week = 29% Delivery >37 Week = 34%	Delivery <37 Week = 0.86 Delivery >37 Week = 0.85	Not reported	N/A	Stepwise Backward Selection	Not reported	Not reported
Carter, et al 2020 (19)	Algorithm	Separate External Validation	N/A	Yes	Not reported	0.731 (0.630,0.831)	Yes	82.6 %(75.4%, 88.6%)	62.7 %(59.6%, 65.6%)	No	N/A	AIC & BIC	N/A	Sensitivity, Specificity, PPV, NPV, LR+, LR-
Celik et,al, 2008 (20)	The Model Developed Using Maternal Data By Selecting Variables Backward Stepwise Logistic Regression Using The Wald Test As Robust Standard Errors Were Estimated	AUC	Splinting	No	Not reported	0.650(NR,NR)	Yes	< 28 Weeks= 80.6% 28-30 Weeks=58.5% 31-33 Weeks=53.0% 34-36 Weeks=28.6%	Not reported	Yes	Complete Case Analysis	Stepwise Backward Selection	Kept Linear	Regression Coefficients
Crane and Hutchens 2008 (21)	The Model Was Developed By ANOVA For Continuous Variable And Logistic Regression For Ordinal Variables	AUC	Not reported	No	Not reported	Not Reported	Yes	63.6 %(33.6%, 87.2%)	77.2 %(67%, 77.5%)	No	N/A	Not reported	Kept Linear	ROC Curve
Damaso et,al, 2019 (22)	The Model Developed From Performance Of A Previously Published Algorithm For First-Trimester Prediction Of Spontaneous Preterm Birth	AUC	N/A	Yes	Not reported	0.67(0.56,0.78)	Yes	60.9%	63.2%	Yes	Complete Case Analysis	Not reported	Kept Linear	ROC Curve Sensitivity Specificity

He et al., 2018 (23)	The Prediction Model With Preterm Birth As The Event And Gestational Age At Birth As The Time Scale. Model-1 Based On Pre-Pregnancy Factors. Model-2 Utilized Pre-Pregnancy Factors And Pregnancy Conditions. The Variables Retained In The Final Models Were Selected Using Backward Elimination.	AUC Harrell C-And D –Statistic	Splinting	Yes	P-Value => 0.05	0.45(NR, NR)	No	Not reported	Not reported	Yes	Multiple Imputation	Backward Elimination	Kept Linear	Prediction Rule
Hebbar and samjhana 2006 (24)	The Model Was Developed At Cut Off Value Of 25mm For The Cervical Length Measurements	AUC, Sensitivity, Specificity NPV And PPV	Not reported	No	Not reported	Not reported	Yes	77%	95%	Not reported	N/A	Not reported	Not reported	Not reported
Hebert et al., 2022 (25)	The Data Were Randomly Split Into Developmental And Validation Datasets. The Pre-Pregnancy Model Could Be Evaluated Prior To Pregnancy. The End Of First Trimester Model Evaluated By The End Of The First Trimester (13weeks). The Mid Pregnancy Model Evaluated By 20 Weeks Of Gestation.	Calibration (Hosmer-Lemeshow Goodness Of Fit) AUC	Not reported	No	Development P-Value = 0.33 Validation P-Value = 0.27	0.66(NR, NR)	No	N/A	N/A	Not reported	N/A	Not reported	Kept Linear	Point Of Care Tool
Newman et al. (26)	The Model Developed By Collecting Data From Pregnant Women And Use Discriminations And Sensitivity Analysis Was Done	AUC	Not reported	No	Not reported	0.68(0.62,0.75)	Yes	13.4% (8%, 20.6%)	97.7 %(97%, 98.2%)	Not reported	N/A	Not reported	Not reported	ROC Curve

Hellemans et al., 1995 (27)	As a Predictor For Preterm Delivery, Cervical Fetal Fibronectin Detection Were Used	Sensitivity Specificity PPV NPV	Not reported	No	Not reported	Not reported	Yes	60%	85%	Not reported	N/A	Not reported	Categorized	Not reported
Fergus et,al, 2013 (28)	The Model Was Developed By Machine Learning Data And Evaluated By ROC Curve, Sensitivity, And Specificity	AUC Sensitivity Specificity	Cross-Validation	No	Not reported	0.95(NR,NR)	Yes	96%	90%	Not reported	N/A	ROC Curve	N/A	AUC Sensitivity Specificity
Jelliffe-Pawlowski et al., 2018 (29)	The Model Was By Dividing Data In Development And Testing Data Set. When Considered In Combination Using The Linear Discriminate For PTB With No Or Presence Of Preeclampsia, Biomarkers Maternal Age >34 Years And Low-Income Status Were Include	AUC	Not reported	No	Not reported	0.750 (0.676,0.825).	No	Not reported	Not reported	Not reported	N/A	Stepwise Backward Selection	Categorized	Algorism
Goyal et,al, 2015a (4)	Not reported	AUC	Splitting	No	No	0.63(NR,NR)	Yes	29%	80%	Not reported	N/A	Stepwise Selection (Forward Selection Followed By Series Of Backward Selection)	Categorized	Risk Score Chart
Jung et al., 2021 (30)	Baseline Maternal And Obstetric Parameters And Outcomes Were Compared Between Delivery <32 Weeks And Delivery > 32 Weeks. Multivariate Logistic Regression Analyses Were Used And AUC Were Calculated	AUC Sensitivity Specificity	Bootstrapping	Not reported	Not reported	0.86(0.71,1.00)	Yes	80%	70%	Not reported	N/A	Not reported	Kept Linear	Model Formula With Regression Coefficentes

Goyal et,al, 2015b (5)	Use Stepwise Elimination Of Variable To Develop Model	AUC	Splitting	No	Not reported	0.63(NR,NR)	Yes	29%	82%	Yes	Complete Case Analysis Multiple Imputation	Stepwise Selection (Forward Selection Followed By Series Of Backward Selection)	Categorized	Risk Score
Nikolova , et al.2018 (31)	The Model Develop By Adding Incremental Value Of Biomarkers	Sensitivity Analysis	Not reported	No	Not reported	Not Reported	Yes	53.9%(37.2%,69.9%)	97.4%(95.1%,98.8%)	Not Sated	N/A	Not reported	Kept Linear	Regression Coefficients
Alleman,et al 2013 (32)	The Model Develop Using Maternal Characteristics And Biomarkers	Sensitivity, Specificity, PPV, NPV And AUC	Bootstrapping	No	No Stated	0.69(NR, NR)	Yes	17.5%	97.0%	Not Sated	N/A	Stepwise Selection (Forward Selection Followed By Series Of Backward Selection)	Not reported	Regression Coefficients
Anderson, et al 1990 (33)	The Model Develops By Examination Of Ultrasonography For Cervical Length Measurement	Sensitivity Specificity	Not reported	Not reported	Not reported	Not Reported	Yes	ABDCX = 46%, VAGCX= 76% MANCX= 71%	ABDCX = 53%, VAGCX= 59% And MANCX= 58%	Not reported	N/A	Logistic Regression	Not reported	Not reported
Grobman et,al, 2016 (34)	The Model Developed By Measuring Cervical Length	AUC	Not reported	Not reported	Not reported	0.63(0.58,0.63)	No	Not reported	Not reported	Yes	Complete Case Analysis	Not reported	Kept Linear	Prediction Rule
Park et al., 2021 (35)	The Bacteria Were Collected From Cervico-Vaginal Fluid After Preterm Birth And Developing Bacterial Risk Score To Predict Preterm-Birth Then	AUC Sensitivity Specificity	Not reported	Not reported	Not reported	0.723(NR, NR)	Yes	71%	59%	Not reported	N/A	Not reported	Kept Linear	Prediction Rule
Pasquini et al.,2014 (36)	Trans-Vaginal Sonographic Assessment Of The CL In Second Trimester Ultrasound Test.	AUC Sensitivity, Specificity PPV NPV LLR	Not reported	Not reported	Not reported	0.657(NR, NR)	Yes	73.9%	45.7%	Not reported	N/A	Not reported	Kept Linear	Prediction Rule

Posthumus et al., 2016 (37)	The Model Was Developed From Maternal Medical And Non-Medical Risk Factors	Calibration Plot	Not reported	Not reported	Not reported	Not Reported	No	Not reported	Not reported	Yes	Multiple Imputation	Stepwise Selection (Forward Selection Followed By Series Of Backward Selection)	Not reported	Risk Score Card
Dziadosz et,al, 2016 (38)	Model Develops Using Linear Regression Model.	Sensitivity Specificity	Not reported	Not reported	Not reported	0.78(0.71,0.81)	Yes	80% (71%,88%	53% (52%,54%)	Yes	Complete Case Analysis	Stepwise Selection (Forward Selection Followed By Series Of Backward Selection)	Kept Linear	Regression Coefficient
Prema and pushpalatha ,2019 (39)	The Model Was Developed By Using Logistic Regression, SVM And Class Imbalance Problem By Using Data Mining Techniques	AUC Sensitivity Specificity	Not reported	Not reported	Not reported	Not reported	Yes	70.%37 For Logistic 83.%87 =For SVM With Linear Kernel 70.4%=SVM With Radial Kernel	79.79%= For Logistic, 72.58 %=For SVM With Linear Kernel ,0.7551=SVM With Radial Kernel	No	N/A	Not reported	Categorized	Prediction Rule
Garcia –blanco et,al, 2017(40)	The Model Was Developed By Nomogram	AUC	Not reported	No	Not reported	0.63(NR, NR)	No	Not reported	No Stated	Yes	Regression Imputation	Not reported	Kept Linear	Nomogram
Raba and Kotarski ., 2016 (41)	By Using All Variable Selection Methods Forward, Back Ward And Step Wise Variables Was Selected And 5 Variables Were Significant	AUC Sensitivity Specificity	Not reported	Not reported	Not reported	0.79(0.75,0.82)	Yes	Not reported	99.5%	Yes	N/A	Stepwise Selection (Forward Selection Followed By Series Of Backward Selection)	Categorized	Algorism
Rafael et al. , 2012 (42)	The Model Was Developed From Previous Twin Preterm Birth Vs Term Birth To Predict Singleton Preterm Birth	AUC	Not reported	Not reported	Not reported	0.77(NR,NR)	Yes	77.8%	73.4%	No	N/A	Not reported	Kept Linear	Regression Coefficients
Crane et,al,	The Model Develops From Biomarkers For	Sensitivity Specificity	Not repo	No Sta	Not reporte	Not Reported	Yes	77.8 %(43.1%,96.1	80.2%(72.7%,86.3 %)	Yes	Complete	Not reported	Kept Linea	Model Formula

1999 (43)	Impacts To Preterm Birth		rted	ted	d			%)			Case Analysis		r	With Regression Coefficients
Baer et al 2018 (44)	The Original Sample Is Divided Into Two As Training And Testing (2/3 And 1/3), Where The Testing Is To Make Validation	AUC	Splinting	Not reported	Not reported	0.591(0.589, 0.594)	No	Not reported	Not reported	No	Not reported	Stepwise Backward Selection	Kept Linear	Risk Score Chart
Riboni et al. ,2011 (45)	The Model Was Developed For Women Below 34 Weeks Of Gestation, Below 37 Weeks Of Gestation And Within 7 Days Delivery	Sensitivity Specificity	Not reported	Not reported	Not reported	Not reported	Yes	Phigfbp-1 Test <37 Weeks = 52.9 %<br>Ffn Test =50%	Phigfbp-1 Test <37 Weeks =89.2 % Ffn Test =81.9%	No	N/A	Stepwise Forward Selection	Kept Linear	Regression Coefficients
Banos et al, 2018 (46)	Model Was Evaluated by AUC, Sensitivity and Specificity	AUC Sensitivity Specificity	Not reported	No	Not reported	For CCI = 0.84(0.75, 0.93)<br>For CL = 0.68(0.56,0.81)	Yes	For CCI = 77.3%,<br>CL = 72.7 %	For CCI = 82.7%, CL = 61.2%	No	N/A	Multivariable Logistic Regression	Not reported	Regression Coefficient
Riboni et al. ,2012 (47)	The Model Was Developed Based On 491 Pregnant Women By The Above 4 Predictors	AUC Sensitivity Specificity	Not reported	Not reported	Not reported	Not reported	Yes	Phigfbp-1=54.1<br>IL-6=22.9%<br>CRP=33.3%<br>Phigfbp-1 + IL-6 + CR=17.9%	Phigfbp-1=72.1%<br>IL-6=94.2 %<br>CRP=86.1 %<br>Phigfbp-1 + IL-6 + CR=99.5%	No	N/A	Stepwise Forward Selection	Categorized	Regression Coefficients
Kim et al., 2011 (48)	The Diagnostic And Prognostic Accuracy Of Each Marker Was Evaluated, And The Optimal Cut-Off Values Of Each Were Selected. Survival Analysis Was Performed To Assess The Relationship Between The Time From Admission To Delivery And The Result Of Each Marker. .	AUC Sensitivity Specificity	Not reported	Not reported	Not reported	0.822(0.748,0.895)	Yes	For Neutrophil 40.2%, Lymphocytes 39.2%, Basophils 49% Monocyte 31.4% Eosinophil 19.6%, CRP 58.8%NLR 52% , Cervix Length 61.2%,Combined Marker 64.2%	Neutrophil 0.89% Lymphocyte 84.9% Basophil 68.5%, Monocyte 82.2%, Eosinophil19.6% CRP 69.9%, NLR 78.1%, Cervix Length 80.0%, Combined Marker 88.3%.	No	N/A	Not reported	Kept Linear	Regression Coefficients
Balardi , et al 2016 (49)	The Model Develops From Maternal Factors	AUC, Sensitivity Specificity	Cross Validation	Not reported	Not reported	0.65 (0.595, 0.986)	Yes	80.5%	70%	No	N/A	Multivariate And Univariate Statistical Data Analysis	Kept Linear	Not reported

Ridout et al., 2019 (50)	The Model Was Developed From Assessing Cervical Length and Quantitative Fetal Fibronectin	AUC Sensitivity	Not reported	Not reported	Not reported	Cervical Length 0.59(0.55,0.64), -For Quantitative Fetal Fibronectin 0.58(0.49, 0.68)	Yes	For Cervical Length 20.3% And 15.2% For <34 And 37 Weeks Respectively	Not reported	No	N/A	Not reported	Kept Linear	Algorism
Gioan et,al, 2018 (51)	The Model Develops From Maternal Characteristics By Adding Factors	AUC, Sensitivity And Specificity	Bootstrapping	No	Not reported	0.77(0.72,0.81)	Yes	74% (63.2%,83.6%)	72.7% (67%,78%)	Yes	Complete Case Analysis	Stepwise Selection (Forward Selection Followed By Series Of Backward Selection)	Kept Linear	Nomogram
Koivu and sairanen, 2020 (52)	Using Determinants Models Logistic Regression (LR), Gradient Boosting Decision Tree (GBDT) And Two Artificial Neural Network (ANN) Models Were Used In This Study Were Developed.	AUC	Not reported	Yes	Not reported	0.64(0.64, 0.64).	No	Not reported	Not reported	No	N/A	Stepwise Forward Selection	Kept Linear	Algorism
Barinov et al , 2021 (53)	The Model Develops From Phosphorylated Insulin-Like Growth Factor Binding Protein-1 (Phigfb-1), Placental Alpha Macroglobulin-1(PAMG-1)	AUC Sensitivity Specificity	Not reported	No	Not reported	Not reported	Yes	Singleton Pregnancy: Phigfbp-1 = 55.6% And PAMG-1 = 97 AND Multiple Pregnancy: Phigfbp-1 = 33.3% And PAMG-1 = 33.3%	Singleton Pregnancy: Phigfbp-1 = 97% And PAMG-1 = 100 % AND Multiple Pregnancy: Phigfbp-1 = 93.3 %And PAMG-1 = 96.6%	No	N/A	Not reported	Kept Linear	Not reported
Kuhrt et al, 2016 (54)	Of The Predictors Considered, Only Quantitative Fibronectin And Previous PTB/PPROM The Best Parametric Survival Model Determined By Lowest AIC And BIC)	AUC Sensitivity Specificity PPV NPV	Splinting	No	P-Value 0.38 Training And For Validation 0.64	0.89(0.77,0.90)	Yes	For Training 66.7 %(44.7%, 84.4%) And For Validation 72 %(50.6%, 87.9%)	For Training 78.9 %(71.9%, 84.9%) And For Validation 77.2 %(70.1%, 80.4%)	No	N/A	Stepwise Forward Selection	Kept Linear	Not Sated
Rohlfing et al.	The Model Was Developed From	AUC	Not repo	No t		0.601 (NR,NR)	No	Not reported	Not reported	No	N/A	Not reported	Kept Linea	Regression

,2020 (55)	Maternal Cardio Vascular Risk Factors(HTN, DM, Cholesterol, Smoking)		rted	reported									r	Coefficients
Bartnicki et al, 1996 (10)	The Model Develops By Fibronectin To Predict LBW	Sensitivity Specificity	Not reported	Not reported	Not reported	Not reported	Yes	67.5%	90.3 %	No	N/A	Stepwise Selection (Forward Selection Followed By Series Of Backward Selection)	Kept Linear	Regression Coefficients
Bastek et al , 2012 (56)	The Model Is Developed From Pregnant Women Who Are Already Presented With Preterm Labor If They Deliver Within 10 Days Of Their Presentation Or Before 37 Weeks Of Gestation (Both Are Preterm)	AUC, Sensitivity Specificity	Bootstrapping	No	Not reported	0.73(NR,NR)	Yes	For Delivery Within 10 Days=84% And For Delivery Before 37 Weeks = 79%	For Delivery Within 10 Days= 51% And For Delivery Before 37 Weeks=50%	No	N/A	Univariable Followed By Multivariable Logistic Regression	Categorized	Regression Coefficients
Saade et al. , 2016 (57)	N/A	AUC Sensitivity Specificity	N/A	Yes	Not reported	0.67 (0.52, 0.81)	Yes	75%	74%	No	N/A	Not reported	Not reported	Regression Coefficients
Lee et al., 2011 (58)	The Bayesian Filtering Algorithm Was Developed.	Sensitivity, Specificity PPV ,NPV	Not reported	No	No	Not Reported	Yes	68.80%	85%	No	N/A	Stepwise Forward Selection	Kept Linear	Algorism
Arabi Belaghi et al, 2021 (59)	The Model is A Machine Learning Based On Logistic Regression Which Utilized A Huge Sample Size By Splitting 2/3 To Model Development and The Remaining 1/3 To The Model Validation	AUC Sensitivity, Specificity	Cross-Validation	No	Not reported	0.65(0.63,0.66)	Yes	During 1st Trimester;62% (47.8%,52.4%) During 2nd Trimester: 66%(60.0%,63.4%)	During 1st Trimester: 64.5% (63.1%,65.4%) During 2nd Trimester: 87.0%(85.5%,88.4%)	Yes	Multiple Imputation	Stepwise Selection (Forward Selection Followed By Series Of Backward Selection)	Categorized	Regression Coefficients
Schmitz et al. , 2008	The Model Was Developed By Classifing Bishop	AUC Sensitivity Specificity	Not reported	Not rep	Not reported	bishop Score 0.848(NR, NR) Sonographic Cervical Length 0.813(NR,NR)	Yes	For Bishop Score=97 % (84%, 100%)	For Bishop Score; 96% (93%, 98%)	No	N/A	N0t Stated	Categorized	Algorism

(60)	Score>4 And >8 Plus Preterm Delivery With In 48 Hours And With In 7 Days			orted				For Cervical Length=95% (75%, 100%)	For Cervical Length=29% (22%, 36%)					
Lee et al., 2020 (61)	Model Was Developed Using Logistic Regression And AUC Was Reported For The Nomogram Of Early Preterm And Late Preterm Prediction.	AUC	Not reported	Not reported	Not reported	0.717 (0.675,0.759)	No	Not reported	Not reported	No	N/A	Stepwise Selection (Forward Selection Followed By Series Of Backward Selection)	Kept Linear	Nomogram; Decision Tree
Menon et al ,2014 (62)	It Is A Machine Learning Based On Pregnant Women Recruited On A Case Control Study	AUC	Cross Validation	Not reported	Not reported	In African Americans: 0.86(NR, NR)<br>In Caucasians: 0.68(NR, NR)	No	Not reported	Not reported	No	N/A	Multivariate Adaptive Regression Splines	Kept Linear	Regression Coefficients
Lee et al., 2015 (63)	The Model Develops From Biomarkers	AUC	Cross Validation	Not reported	Not reported	0.852 (0.766, 0.938)	No	Not reported	Not reported	No	N/A	Stepwise Forward Selection	Kept Linear	Not reported
Leow et al., 2020 (64)	Prospective Cohort Was Conducted And Seven Predictors Were Identified Then AUC And Sensitivity Specificity Analysis Was Done Finally Model Was Presented Using Algorithm	AUC Sensitivity, Specificity	Splinting	Not reported	Not reported	0.88 (NR, NR)	Yes	For Development 100%(76%, 100%)And 91%(62%, 100%) For Validation Set	74% (66%, 81%) development and 78%(70%,84%)For Validation	No	N/A	Not reported	Kept Linear	Algorism
Cohen et al ,2014 (11)	The Model Develops From Pregnancy Associated Proteins	AUC Sensitivity, Specificity	Not reported	Not reported	Not reported	0.72 (NR,NR)	Yes	63%	71%	Yes	Complete Case Analysis	Univariable And Multivariable Logistic Regression	Kept Linear	Regression Coefficients
Leung et al., 2005 (65)	Model Developed Using Cervical Length And Funneling Then Logistic Regression Was Conducted Model Performance Was Measured Using	Sensitivity Specificity PPV NPV LR+	Not reported	Not reported	Not reported	0.56 (0.51,0.62)	Yes	Less Than 27mm Cervical Length With Funneling =26.3 %(6.5%, 46.1%)	99 %(98.6%, 99.4%)	No	N/A	Not reported	Categorized	Not reported

	Sensitivity Specificity Analysis.												
Lin et al., 2021 (66)	The National Vital Statistics System (NVSS) Was Queried For Singleton ART-Treated Pregnant Women From Multivariable Cox Regression Was Used To Develop The Early (< 32 Weeks) Or Late (< 37 Weeks) PTB Risk Model Using Both Statistical Significance. Cox-Proportional Model Was Used And The Bootstraps Using 1000 Repeats Were Used For Internal Validation	AUC Calibration Slope Brier Score	Bootstrapping	Calibration Slop 0.99	Not reported	0.684(NR,NR)	No	Not reported	Not reported	No	N/A	Stepwise Forward Selection	Categorized	Nomogram
Liu et al., 2020 (67)	Model Was Developed Using Original Beta Coefficients And Presented With Nomogram And Formula	AUC Sensitivity Specificity	Bootstrapping	Not reported	P-Value 0.02	0.803(0.76,0.85)	Yes	65.80%	84%	No	N/A	Not reported	Categorized	Nomogram
Van de Mheen et al .,2014 (68)	The Model Was Developed From The Previous RCT Trial For AMPHA Drug Trial For Predicting Preterm Birth	Calibration Plot, Sensitivity Specificity	Splitting	Not reported	Calibration was good	0.68 (0.63 ,0.72)	Yes	AT Cervical Length Cut Off Value>40 Mm=88.4%	At Cut Off Value Of CL > 40 =30.5	No	N/A	Not reported	Categorized	Regression Coefficients
Yazici et al. ,2004 (69)	The Model Was Developed By Comparing Trans Vaginal And Trans Peroneal Sonography To Predict Preterm Delivery By Examining At 24 Weeks Gestation	AUC Sensitivity	Not reported	Not reported	Not reported	For trans vaginal Sonography; 0.801(NR,NR)<br>Trans Perennial Sonography; 0.857(NR,NR)	Yes	Trans Vaginal Sonography=72 % Trans perennial Sonography= 77%	Not reported	No	N/A	Not reported	Kept Linear	Algorism

Mueller-HeubachGuzick., 1989 (70)	This Study Was Evaluation Of Previously Developed Scoring System	Sensitivity	N/A	Yes	Not Sated	Not reported	Yes	28.80%	Not reported	No	N/A	Not reported	Categorized	Model Formula With Regression Coefficients
Mortensen et al.,1987 (71)	Sensitivity, Specificity And Predictive Values Reported Using Four Comparison Groups	Sensitivity, Specificity PPV NPV	Not reported	No	Not reported	Not reported	Yes	For Past Medical History=42% ; For With Complication In Current Pregnancy 71% And For The Group With Cervical Screening 0.33	For Past Medical History =82% For With Complication In Current Pregnancy70% and For Cervical Screening 88%	No	N/A	Not reported	Categorized	Not reported
Morken et al., 2014 (72)	Model Was Developed And Validated Internally. The AUC Of Each Model Was Reported. The Model Developed Was Through Multivariable Logistic Regression.	AUC	Cross Validation	Not reported	Not reported	0.74 (0.73, 0.740)	No	Not reported	Not reported	No	N/A	Stepwise Forward Selection	Kept Linear	Model Formula With Regression Coefficients
Zhang et al . ,2021 (73)	The Model Was Developed By Comparing Of Two Nomogram Between 22-24 And 26-28 Weeks Gestation To Predict Preterm Birth	Calibration Plot , AUC ,Sensitivity Decision Curve Analysis	Bootstrapping	Not reported	C-statistics Nomogram1=0.870 For Nomogram-2=0.908	0.881(NR,NR)	Yes	For Nomogram-1= 80 %And For Nomogram-2 =69%	For Nomogram-1 =75 % For Nomogram-2 =88.6%	No	N/A	Stepwise Selection (Forward Selection Followed By Series Of Backward Selection)	Keep Linear	Nomogram
Macdonald – wallis et,al 2015 (74)	The Model Develops From Maternal Factors	AUC, Calibration Plot Sensitivity Specificity	Cross Validation	Not reported	Calibration Plot	0.63(NR,NR)	No	95%	37 %(31%, 42%)	Yes	Multiple Imputation	Not reported	Categorized	Score Chart
Mailath	The Model Develops	Calibration	Not	Ye	(P	0.80(0.70,0.81)	No	Not reported	Not reported	Yes	Comp	Not reported	Kept	Nomogra

-pokornyet,al, 2015 (75)	From PROM, Cervical Length, CRP	Plot	reported	s	Value = 0.39)						lete Case Analysis		Linear	m
McLean et,al, 1999 (76)	The Model Develops From Concentrations Of Corticotrophin-Releasing Hormone. Î±-Fetoprotein	AUC	Not reported	Not reported	Not reported	0.37(NR,NR)	No	Not reported	Not reported	Yes	Complete Case Analysis	Not reported	Kept Linear	Regression Coefficients
Watson et al. ,2020 (77)	The Model Was Developed From Secondary Data Analysis From Previous Cohort By Using Cervical Length Fetal Fibroectin	AUC Calibration Plot	Not reported	Not reported	Calibration Plot	For Quantitative fibronectin Validated=0.90 (NR,NR) For Cervical Length 0.87(NR,NR)	No	Not reported	Not reported	No	N/A	Not reported	Kept Linear	Algorism
Mehta-Lee et,al, 2017 (78)	The model develop from maternal factors	AUC	Not reported	Not reported	Not reported	0.647(NR,NR)	No	Not reported	Not reported	Yes	Complete case analysis	Not reported	Kept linear	Nomogram
Mendoza et,al, 2021 (79)	The model develops from by cervical pessary placement	AUC	Not reported	Not reported	Not reported	0.88(0.76,1.00)	Yes	100%	80%	Yes	Complete case analysis	Not reported	Not reported	Prediction rule
Zhu et al . ,2017 (80)	It was developed fro maternal and fetal characteristics	sensitivity Specificity	Not reported	Not reported	Not reported	Not reported	Yes	82.6%	88.1%	No	N/A	Not reported	Categorized	Regression coefficients
Radhouane et al. ,2017 (81)	The model was developed by adding cervical length measurement at 12-14 weeks and 22-24 weeks gestation compared to digital assessment of cervix by vaginal touch at first and second trimester of pregnancy's	sensitivity Specificity	Not reported	Not reported	Not reported	Not reported	Yes	82.5%	78.7%	No	N/A	Not reported	Kept linear	Regression coefficients
Bachkangi et	The model is developed to	AUC, sensitivity	Not repo	No t	Not reporte	AEA = 0.707(NR,NR) OEA = 0.628(NR,NR)	Yes	AEA = 67% OEA = 44%	AEA = 67% OEA = 62%	no	N/A	univariable analysis	Kept linear	Regression

al ,2019 (82)	determine if plasma concentrations of some biomarkers increase among women who are at higher risk of preterm birth	specificity	rted	reported	d							followed by multivariable analysis		coefficients
Szymusik et al. ,2011 (83)	The model was developed by identifying spontaneous singleton pregnancies and Invitro fertilization singleton pregnancies to predict preterm birth	sensitivity Specificity	Not reported	Not reported	Not reported	Not Reported	Yes	75%	91.7%	No	N/A	Not reported	Categorized	Regression coefficients
Schaaf et al. ,2012 (84)	The model was developed using nationwide perinatal registry data to predict preterm birth	AUC, sensitivity calibration plot	Bootstrapping	Not reported	Calibration plot	0.63(0.63 0.63)	Yes	42%(42%,42%)	93%(93%,93%)	yes	Complete case analysis	Stepwise backward selection	Categorized	Regression coefficients

Table S4: Study characteristics prediction models for still birth

Study	Country	Study Design	Center	Study Population	Outcome	Sample Size		Outcome(N)	Incidence	Events Per Variable	Number Of Predictors	Model Type	Model Analysis
						Development	Validation						
Akolekar et al, 2016b (85)	UK	Prospective Cohort	Multi-Centered	Pregnant Women Attending For Routine Pregnancy Care At 11-13 Weeks Gestation	Still Birth	45452	Not reported	227	0.49%	Not reported	-Placental Growth Factor (PLGF), Fetal Ductus Venosus Pulsatility Index For Veins (DV-PIV) -Uterine Artery Pulsatility Index (UT-PI)	Incremental Value	Logistic Regression
Goyal et,al, 2015b (5)	USA	Retrospective Cohort	Multi Centered	All Pregnant Women	Stillbirth	263	146	1	0.4%	0.14	Multiparous, Race, Rape, Other Medical Conditions, Primi-Parous, Prenatal Care Entry, Prior Preterm Birth	Derivation	Logistic Regression
Akoleka	UK	Prospectiv	Multi	Women	Stillbirth	70,003	Not	268	0.38%	Not reported	Maternal Factor, HC,	Increm	Logistic

r, 2016a (86)		e Screening	Centered	Attending ANC At 19-24 Weeks Gestation			reported				AC,FL Uterine Artery Pulsatility Index (UT-PI)	ental Value	Regression
Amark et al, 2018 (87)	Sweden	Register-Based Cohort S	Not reported	Pregnant Women With Overweight Or Obesity	Still Birth	45,859	Not reported	282	0.0062	Not reported	BMI,, Pregnancy-Associated Plasma Protein-A (PAPP-A), maternal Age, Smoking , Country of birth and Parity	Derivation	Logistic Regression
Aupont et al, 2016 (88)	UK	Prospective Screening	Multi Centered	Women Attending ANC At 19-24 Weeks Gestation	Still Birth	70,003	Not reported	268	0.38%	Not reported	Placental Growth Factor Uterine Artery Pulsatility Index ,HC,AC ,Maternal Factors	Incremental Value	Logistic Regression
Bahado-singh et al, 2019 (89)	UK	Prospective Screening	Single Centered	Pregnant Women First Trimester	Still Birth	180	Not reported	60	0.333	Not reported	Glycine, Acetic Acid, L-Carnitine, Creatine	Derivation	Logistic Regression
Schneuer et al. , 2014 (9)	Australia	Prospective Cohort	Single Centered	Pregnant Women Attending First Trimester	Still birth	4621	Not reported	Not reported	Not reported	Not reported	Angiopoietin 1, Angiopoietin 2, Angiopoietin 1/Angiopoietin 2 Ratio, Maternal Age, Socio-Economic Status ,Country of birth	Derivation	Logistic Regression
Koivu and Sairanen ., 2020 (52)	USA	Cross Sectional	Population Based	Infant Birth And Death Data Sets Containing Pregnancies During The Years CDC, National Center Of Health Statistics	Stillbirth	266,419	Not reported	249	0.1%	Not reported	Maternal age , Race, Marital Status, Education , Number of previous terminations, Special Supplemental Nutrition Program (WIC) , Smoking ,BMI, Height, Weight , Parity, Pre-Pregnancy Diabetes, Gestational Diabetes, Pre-Pregnancy Hypertension, Gestational Hypertension, Hypertension Eclampsia, Prior Preterm Births, Infertility, Assisted Reproductive Technology (ART), Previous Cesarean Sections, other medical condition.	Derivation	Logistic Regression
Mastrodima et,al, 2016 (90)	UK	Prospective Cohort Study	Multi Centered	1st Trimester Pregnant	Still Birth	76629	Not reported	268	0.35%	89.3	PAPP-A, Uta-PI and DV-PIV	Derivation	Logistic Regression

Table S4: Study characteristics prediction models for still birth (continued)

Study	Model Development	Model Evaluation	Internal Validation	External Validation	Calibration (P Value Hosmer-Lemeshow Test Or Calibration Plot)	Model Discrimination (AUC With 95% CI)	Model Performances	Sensitivity With 95%CI	Specificity With 95%CI	Handling Of Mission Value	Method Of Handling Missing Value	Model Selection	Handling Of Continuous Data	How Is The Model Presented
Akolekar et al, 2016b (85)	Not reported	AUC	Not reported	No	Not reported	0.852(0.816,0.888)	No	Not reported	Not reported	Not reported	N/A	Not reported	Not reported	Regression Coefficients
Goyal et,al, 2015b (5)	Use Stepwise Elimination Of Variable To Develop Model	AUC	Splitting	No	Not reported	0.63(NR,NR)	Yes	29%	82%	Yes	Complete Case Analysis Multiple Imputation	Stepwise Selection (Forward Selection Followed By Series Of Backward Selection)	Categorized	Risk Score
Akolekar, 2016a (86)	The Model Develops Based On UT-PI And When Other Predictors Are Added (HC, AC, FL And Maternal Factors) In Combination	AUC	Not reported	Not reported	Not reported	0.748(0.711, 0.785)	No	Not reported	Not reported	No	Not reported	Univariate Logistic Regression	Not reported	Regression Coefficients
Amark et al, 2018 (87)	Model Was Develops From Maternal Characteristics And With AUROC Curve, Sensitivity And Specificity	AUC Sensitivity Specificity	Cross Validation	No	Not reported	0.69(0.64,0.74)	Yes	28%	90%	Yes	Complete Case Analysis	Univariable Logistic Regression Followed By Multivariable Logistic Regression	Not reported	Regression Coefficients
Aupont et al, 2016 (88)	This Model Is Intended To Test The Incremental Value Of Placental Growth Factor (PLGF) On Prediction Of Stillbirth	AUC	Not reported	No	Not reported	0.950(0.932,0.967)	No	Not reported	Not reported	No	N/A	Not reported	Not Stead	Regression Coefficients
Bahado-singh et al,	The Model Is Developed To Identify Metabolites Which Predict	AUC, Sensitivity Specificity	Not reported	No	Not reported	0.860(0.793,0.927)	Yes	For Metabolites = 66.7% When	For Metabolites = 62.5%, SP When	No	N/A	Univariable Followed By Multivariable Logistic	Not reported	Regression Coefficients

2019 (89)	Stillbirth							Combined With Clinical Predictors = 68.3% For Liquid Chromatography Coupled With Mass Spectrometry Combined With Clinical Predictors = 81.8%	Combined With Clinical Predictors = 68.3%, For Liquid Chromatography Coupled With Mass Spectrometry Combined With Clinical Predictors = 75.8%			Regression		
Schneuer et al. , 2014 (9)	The Model Was Developed From Maternal Characteristics Like Age, Weight, Country Of Birth, Socio Economic Status With The Above Predictors	AUC Sensitivity Specificity	Not reported	Not reported	Not reported	Ang-1/Ang-2 Ratio ; 0.70 (NR,NR) Maternal Risk Factors 0.58(NR,NR)	Yes	<10%	Not reported	Yes	Multiple Imputation	Not reported	Not reported	Algorism
Koivu and Sairanen., 2020 (52)	Using Determinants Models Logistic Regression (LR), Gradient Boosting Decision Tree (GBDT) And Two Artificial Neural Network (ANN) Models Were Used In This Study Were Developed.	AUC	Not reported	Yes	Not reported	0.61(0.56, 0.66	No	Not reported	Not reported	No	N/A	Stepwise Forward Selection	Kept Linear	Algorism
Mastrodima et,al, 2016 (90)	The Develops From PAPP-A, UTA-PI And DV-PIV	AUC	Not reported	Not reported	Not reported	0.657(0.621, 0.693)	No	Not reported	Not reported	Yes	Complete Case Analysis	Not reported	Categorized	Regression Coefficients

Table S5: Study characteristics prediction models for small for gestational age

Study	Country	Study Design	Center	Study Population	Outcome	Sample Size		Outcome(N)	Incidence	Events Per Variable	Number Of Predictors	Model Type	Model Analysis
						Development	Validation						
Kim et al., 2019 (91)	Korea	Retrospective Follow Up	Single Centered	Pregnant Women	SGA	442	Not reported	114	25.79%	22.8	BPD, HC, AC, FL ,EFW	Derivation	A Linear Mixed Model
Antunes et al,2021 (92)	Brazil	Retrospective Cohort	Single Centered	Pregnant Women Between 11 And 14 Weeks Of Gestation	SGA	615	Not reported	44	7.1%	Not reported	-Family History of SGA, Pregnancy-Associated Plasma Protein, Placental Growth Factor, Uterine Artery Mean Pulsatility Index, Mean Arterial Pressure	Derivation	Logistic Regression
Poon et al., 2015 (93)	London	Prospective Observational	Multi Centered	Second Trimester Of Pregnancy	SGA	7816	Not reported	389	5%	Not reported	BMI, maternal Age, Parity, Race , Mode of conception, Gestational age, Fetal Biometry, Birth Weight ,Uterine Artery Pulsatility Index, Placental Growth Factor	Incremental Value	Logistic Regression
Posthumus et al., 2016 (37)	Netherland	Prospective Cohort	Multi Centered	All Pregnant Women	SGA	836	Not reported	101	12%	Not reported	Previous history of ABO, Smoking, Alcohol Drinking, Drug Abuse , Social Support, Family Income, Housing Condition	Derivation	Logistic Regression
Baekgaard Thorson et al ,2020 (94)	Denmark	Prospective Cohort	Population Based	Pregnant Women	SGA	1937	Not reported	44	2.3%	Not reported	Placental Growth Factor	Derivation	Logistic Regression
Kato et al., 2021 (95)	Brazil	A Cross-Sectional Retrospective	Population Based	Singleton Pregnancy	SGA	231	Not reported	42	18.25	42	Ultrasonography	Derivation	Not reported
Quant et al.,2016 (96)	USA	Prospective Cohort	Single Centered	Women With Singleton Gestations 11 And 14 Week Gestation	SGA	373	Not reported	36	9.7%	Not reported	Placental Volume, Placental Quotient, Mean Placental Diameter, Mean Chorionic Diameter,	Derivation	Logistic Regression

Quinton et al. ,2015 (97)	Australia	Prospective Observational	Single Centered	Pregnant Women At 28-32 Weeks Of Gestation With Singleton Pregnancies	SGA	41	Not reported	8	20%	Not reported	-HC/AC Ratio HC/FL Ratio HC,AC,FL ,BPD, Umbilical Artery Doppler Pulsatility Index, Four Quadrant Amniotic Fluid Index, EFW	Derivation	Logistic Regression
Leavitt et al., 2021 (98)	USA	Prospective Cohort	Multi Centered	Women Referred For Fetal Growth Ultrasounds Between 26 And 36 Weeks Of Gestation	SGA	199	Not reported	94	47.20%	47	Cerebro-Placental Ratio and Umbilical Artery (UA) Doppler	Derivation	Logistic Regression
Schneuer et al. , 2014 (9)	Australia	Prospective Cohort	Single Centered	Pregnant Women Attending First Trimester	SGA	4621	Not reported	Not reported	Not reported	Not reported	Angiopoietin 1 ,Angiopoietin 2 - Angiopoietin 1/Angiopoietin 2 Ratio, maternal Age, Socio-Economic Status ,Country of birth	Derivation	Logistic Regression
Schwartz et al. , 2014 (99)	USA	Prospective Cohort	Single Centered	Women Carrying Singleton Pregnancies Who Presented At 11-14 Weeks Gestation	SGA	578	578	56	9.7%	Not reported	Placental Volume (PV), ,Placental Quotient [PQ] , Gestational Age, ,Mean Placental Diameter ,Chorionic Diameters, Placental Morphology Index ,Placental Growth Factor and Placental Protein-13	Derivation	Logistic Regression
Schwartz et al. ,2012 (100)	USA	Retrospective Cohort	Single Centered	Singleton Pregnancy Between 18 And 24 Weeks Gestation	SGA	1909	Not reported	Not reported	Not reported	Not reported	Maximal Placental Diameter, Mean Placental Diameter ,Maximal Placental Thickness, AC , BPD , BPD; EFW Lag, Estimated Fetal Weight; , Femoral length	Derivation	Logistic Regression
Lesmes et al., 2015(b) (101)	UK	Prospective Follow Up	Single Centered	Women With Second Trimester Of Pregnancy At 19+0 To 24+6 Weeks Gestation	SGA	9715	Not reported	481	5%	96.2	Serum Pregnancy Associated Plasma Protein-A, Human Chorionic Gonadotropin With Or Without Fetoprotein (AFP), Serum Placental Growth Factor (PlGF) and Serum Soluble FMS-Like Tyrosine Kinase-1 (Sflt-1)	Derivation	Logistic Regression
Seravalli et al. ,2014 (102)	USA	Prospective Follow Up	Multi Centered	Second Trimester Pregnant	SGA	1982	Not reported	172	8.6%	Not reported	AC,HC,EFW Uterine Artery, Umbilical Artery Pulsatility Index;	Derivation	Logistic Regression

Lesmes et al., 2015(a) (103)	UK	Prospective Follow Up	Single Centered	Women Attending For Their Routine Hospital Visit In The Second Trimester Of Pregnancy	SGA	88,187	Not reported	5003 (598 And 4,405 Delivering At <37 And >37 Weeks Gestation Respectively)	5.70%	Not reported	Maternal Height , Cigarette Smoking, Assisted Conception, Race, Parity, Inter-pregnancy interval, Previous PE, Previous SGA ,Previous Both SGA And PE, Chronic Hypertension, Diabetes Mellitus, SLE / APS, HC , AC, FL	Derivation	Logistic Regression
Lesmes et al., 2015(C) (104)	UK	Prospective Follow-Up	Multi Center	Women Attending Their Routine Hospital Visit In The Second Trimester Of Pregnancy	SGA	63 975	63975	3702	5.8%	Not reported	Maternal Factors, Fetal Biometry ,UTA-PI and MAP	Derivation	Logistic Regression
Souka et al. ,2012 (105)	Greece	An Observational Cross-Sectional	Single Centered	Third-Trimester Pregnancies Who Had Ultrasound Examination	SGA	2310	Not reported	121	5.20%	Not reported	BPD, HC,AC ,Femur Length , Umbilical Artery (UA) Pulsatile Index(PI) and ,EFW	Derivation	Logistic Regression
Souka et al. ,2013 (106)	Greece	Observational Cross-Sectional	Multi Centered	Pregnant Women With Ultrasound Scan Examination At 30-40 Weeks	SGA	5978	Not reported	214	5.7%	Not reported	Ultrasonography and Estimated Fetal Weight	Incremental Value	Logistic Regression
MacDonald et al., 2018 (107)	Australia	Nested Case Control	Single Centered	102 Cases Of SGA Infants And A Matched Group Of 207 Controls; And 39 Cases Of Preeclampsia.	SGA	348	Not reported	105	30.17%	35	Placental Growth Factor ,Soluble FMS-Like Tyrosine Kinase 1,Placental Growth Factor to soluble FMS-Like Tyrosine Kinase 1 Ratio	Derivation	Logistic Regression
Ye et al. , 2020 (108)	Japan	Cross Sectional	Single Centered	Threated Preterm Labour And Healthy Group Of Term Labor	SGA	95	Not reported	17	17.80%	Not reported	Anti-Porphyromonas Gingivalis Immunoglobulin G Subclass Antibody	Derivation	Logistic Regression
McCowan et,al, 2017 (109)	Australia	Prospective Cohort	Single Centered	3rd Trimester Pregnant	SGA	3735	1871	633	11.2	301.5	PAPP-A, Ng/Ml, VEGFR1,	Derivation	Logistic Regression
Wen et	USA	Retrospec	Multi	Twin	SGA	116 070	28911	Not reported	Not reported	Not reported	Maternal race, education,	Derivation	Logistic

al., 2005 (110)		tive cohort	centered	pregnancy's women with her neonates							marital status, parity, prenatal care visit initiation, , and cigarette smoking		regression
Rosendahl and Kivinen.,1991 (111)	Finland	Prospective follow up	Single centered	Singleton pregnancies	SGA	1112	Not reported	67	6%	Not reported	Smoking, pre-pregnancy weight, pregnancy associated hypertension, previous SGA infant	Derivation	Logistic regression

Table S5: Study characteristics prediction models for small for gestational age (Continued)

Study	Model Development	Model Evaluation	Internal Validation	External Validation	Calibration (P Value Hosmer-Lemeshow Test Or Calibration Plot)	Model Discrimination (AUC With 95% CI)	Model Performances	Sensitivity With 95%CI	Specificity With 95%CI	Handling Of Mission Value	Method Of Handling Missing Value	Model Selection	Handling Of Continuous Data	How Is The Model Presented
Kim et al., 2019 (91)	A Linear Mixed Model For Repeated Measures Was Used To Examine The Impact Fetal Biometric Parameters Over The Measurement Time. ROC Analysis Was	AUC Sensitivity Specificity PPV And NPV	Not reported	Not reported	Not reported	EFW=0.826(0.778,0.872) AC=0.806(NR,NR)	Yes	EFW=60.6% AC=79.5%	EFW=87.6% AC=71.7%	Not reported	N/A	Not reported	Categorized	ROC Curve
Antunes et al,2021 (92)	The Risk Of Small For Gestational Age Of Lower Than 5th And 10th Percentile Was Predicted Among First Trimester Pregnant Women	AUC Sensitivity, Specificity, PPV, NPV, LR+, LR-	Not reported	Not reported	Not reported	0.676(0.585,0.766)	Yes	SGA< 5th Percentile=30%	SGA< 5th Percentile 90.1% For Both	Not reported	N/A	Univariate And Multivariate Logistic Regression	Not reported	Regression Coefficients
Poon et al., 2015 (93)	The Model Was Developed From Multicenter Study And Adding Maternal Factors From The Previous Study Of Biophysical And	AUC	Not reported	No	Not reported	0.874 (0.882,0.886)	No	Not reported	Not reported	Not reported	N/A	Not reported	Categorized	Prediction Rule

	Biochemical Markers													
Posthumus et al., 2016 (37)	The Model Was Developed From Maternal Medical And Non-Medical Risk Factors	Calibration Plot	Not reported	Not reported	Not reported	Not reported	No	Not reported	Not reported	Yes	Multiple Imputation	Stepwise Selection (Forward Selection Followed By Series Of Backward Selection)	Not reported	Risk Score Card
Baekgaard Thorson et al ,2020 (94)	Model Was Developed By Considering Plasma Growth Factor	AUC Sensitivity, Specificity, PPV And NPV	Not reported	Not reported	Not reported	0.75(NR,NR)	Yes	SGA = 69.7 % Preeclampsia With SGA = 100% Non-Preeclampsia With SGA =64% Term SGA = 66.7%	SGA = 72.4 % Preeclampsia with SGA = 87.3 % Non-Preeclampsia With SGA =66.5%Term SGA = 72.2%	No	Not reported	Multivariate Logistic Regression	Not reported	Regression Coefficentes
Kato et al., 2021 (95)	A Retrospective and Cross-Sectional Study Involving 231 Fetuses	AUC Sensitivity Specificity PPV NPV	Not reported	Not reported	Not reported	0.832(0.766,0.899)	Yes	61.9 %(47.2%,76.6 %)	84.1 %(78.90%, 89.30%)	No	N/A	Not reported	Not reported	Not reported
Quant et al.,2016 (96)	The Model Was Developed From Collected Cohort Done By Done First Trimester and Variables Selected By Multi Variable Logistic Regression	Sensitivity Specificity AUC	Not reported	Not reported	Not reported	0.74(0.72,0.76)	Yes	38 %(32.5%, 45%)	82 %(79.5%, 84.4%)	No	N/A	Based On Clinical Experience And Multi Variable Logistic Regression	Kept Linear	By Prediction Rule
Quinton et al. ,2015 (97)	The Model Was Developed From Variables Quadrant Maternal and fetal factors	AUC Sensitivity Specificity	Not reported	Not reported	Not reported	0.742(NR,NR)	Yes	88%	52%	NO	N/A	Not reported	Kept Linear	Prediction Rule
Leavitt et al., 2021 (98)	This prognostic model compares the predictive accuracy of cerebro-placental ratio (CPR) versus umbilical artery (UA)	AUC Sensitivity Specificity	Not reported	Not reported	Not reported	CPR=0.51(0.48,0.53) and Abnormal UA Doppler= 0.54, (0.50,0.58)	yes	low CPR 4.4% and Abnormal UA Doppler 12.8%	low CPR 96.5 and Abnormal UA Doppler 95.6%	No	N/A	Not reported	Kept linear	Not reported

Schneuer et al., 2014 (9)	The Model Was Developed From Maternal Characteristics Like Age, Weight, Country Of Birth, Socio Economic Status	AUC Sensitivity Specificity	Not reported	Not reported	Not reported	Ang-1/Ang-2 Ratio ; 0.70 (NR,NR) Maternal Risk Factors 0.58(NR,NR)	Yes	<10%	Not reported	Yes	Multiple Imputation	Not reported	Not reported	Algorism
Schwartz et al., 2014 (99)	The Model Was Developed By Using 3D Ultrasound Measurement Of Placental Parameters and Maternal Serum	AUC And Sensitivity Specificity	Bootstrapping	Not reported	Not reported	PV= 0.793(0.723, 0.862) PQ=0.797(0.728 ,0.866) MVD= 0.784 (0.716,0.852) PMI= 0.776 (0.698,0.853) MCD= 0.804 (0.734,0.874) Mean PI=0.717 (O.628,0.807)	Yes	PV; 46.4% (28%,65.8%) PQ;46.4%(8 % ,65.8%) MPD; 39.3% (22.1%59.3 %),PMI; 50.0(31.1%, 68.9%%) MCD; 46.4% (28%,65.8%) Mean PI=28.6% (14%, 48.9%)	PV= 79.1% (75.4%,82.4%) PQ=80.0% (76.4%,83.2%) PMI= 50.0% (31.1%, 68.9%) MCD; 46.4% (28%,65.8%) Mean PI; 28.6 %(14%, 48.9%)	No	N/A	Not reported	Categorized	Algorism
Schwartz et al. ,2012 (100)	The Model Was Developed From Two-Dimensional Sonographic Placental Measurement By Estimating Biometric Lags and Placenta Diameter and Thickness	AUC	Not reported	Not reported	Not reported	Not reported	No	Not reported	Not reported	No	N/A	Not reported	Kept Linear	Prediction Rule
Lesmes et al., 2015(b) (101)	Prospective Screening Study In Singleton Pregnancies, Delivered SGA Neonates With Birth Weight<5th Percentile (SGA <5th), In The Absence Of PE Was Done.	AUC	Not reported	Not reported	Not reported	0.963 (0.958, 0.967)	No	Not reported	Not reported	No	N/A	Not reported	Not reported	Not reported
Seravalli et al. ,2014	The Model Was Developed From Previously Cohort	AUC Sensitivity Specificity	Not reported	Not reported	Not reported	-AC 0.700(NR,NR) - HC =0.628(NR,NR),	Yes	AC =52 %HC=73 %EFW=54%	AC=77 %HC=50% EFW=70%	No	N/A	Not reported	Categorized	Regression Coefficien

(102)	Of First Trimester Pregnancy's Who Had Follow Up Second Trimester Pregnancy's By Incorporating Doppler Indices And Fetal Biometry Markers					- EFW =0.667(NR,NR)<br>Uta = 0.560(NR,NR)<br>UA-PI =0.561(NR,NR)		Uta=41%<br>UA=52%						ts
Lesmes et al., 2015(a) (103)	Model Was Developed Using Multivariable Logistic Regression	AUC	Not reporte d	Not reported	Not reported	0.804(0.802,0.807)	No	Not reported	Not reported	N0	N/A	Not reported	Kept Linear	Model Presented With Regressio n Coefficien ts And Formula
Lesmes et al., 2015(C) (104)	Model Develops From Combined Maternal Factors	AUC	Not reporte d	Not reported	Not reported	0.852 (0.850,0.855)	No	Not reported	Not reported	No	N/A	Not reported	Kept Linear	Regressio n Coefficien ts
Souka et al. ,2012 (105)	The Model Was Developed Previously Cohort Of 1st Trimester Pregnancy's Who Had 3rd Trimester Follow Up By Measuring Fetal Biometry Indices Through Doppler Ultrasound	AUC<br>Sensitivity<br>Specificity	Not reporte d	Not reported	Not reported	-BPD; 0.745(0.7015,0.7883)<br>HC ;0.7509 (0,7074,0.7943)<br>AC;0.8470(0.8136,0. 8803)<br>FL ;0.7371(0.6904,0.7837)<br><br>EFW;0.8657(0.8352, 0.8961)<br>UA-PI; 0.6350(0.583,0.6869)	Yes	At 25 % Coverage BPD =58 % HC=63% AC =74 % FL =60% EFW = 81% And UA-PI Mom =39%	Not reported	No	N/A	Not reported	Kept Linear	Regressio n Coefficien ts
Souka et al. ,2013 (106)	The Model Was Developed By Estimating Fetal Biometry And Ultrasound Scanning In Late 3rd Trimester Of Pregnancy's	AUC<br>Sensitivity	Not reporte d	Not reported	Not reported	0.9074(NR,NR)	Yes	At 25% Coverage 87.9% On Late Scan At 35-37 Weeks By Ultrasound In Early Scan 80.2% In Below 34 Weeks	Not reported	No	N/A	Not reported	Kept Linear	Algorism

MacDonald et al., 2018 (107)	Model Was Developed Using Logistic Regression	AUC Sensitivity Specificity PPV NPV	Not reported	Not reported	Not reported	0.8637(NR,NR)	Yes	24.50%(16.5%,34%)	94.20%(90.1%,97%)	No	N/A	Not reported	Categorized	Not reported
Ye et al. , 2020 (108)	The Model Was Developed From Threated Preterm Labor and Health Groups Of Women That Uses Previously Collected Data At Dental Clinic And Predict SGA From Bacterial Antibody	AUC Sensitivity Specificity	Not reported	No	Not reported	0.792(0.634, 0.951)	Yes	78.6%	72.7%	No	N/A	Not reported	Categorized	Algorism
McCowan et,al, 2017 (109)	The Model Develops From Bio Markers	AUC	Splinting	Not reported	Not reported	0.59(0.56, 0.62)	Yes	19%(14%,24%)	Not reported	Yes	Complete Case Analysis	Not reported	Kept Linear	Regression Coefficients
Wen et al. , 2005 (110)	The model use population based data set has two SGA prediction models for twin pregnancies, one for predicting the twin sets with one or both SGA twins (model 1) and the other for predicting the twin sets with both SGA twins (model 2).	AUC sensitivity Specificity	Not reported	Not reported	Yes	0.604(0.60,0.608)	Yes	Model 1= 52.3%, Model 2= 32.8%,	Model-1 = 62.5%, model-2= 85.4%,	No	N/A	Not reported	Categorized	Regression coefficients
Rosendahl and Kivinen. ,1991 (111)	The model was developed by collected data from women and ultrasonography examination, their combined effect to predict SGA	sensitivity Specificity	Not reported	Not reported	Not reported	Not reported	yes	70.1%	95.50%	No	N/A	Not reported	Kept linear	Regression coefficients

Table S6: Study characteristics prediction models for composite adverse birth outcome

Study	Country	Study Design	Center	Study Population	Outcome	Sample Size		Outcome(N)	Incidence	Events Per Variable	Number Of Predictors	Model Type	Model Analysis
						Development	Validation						
Ngwenya. et al, 2021 (112)	Zimbabwe	A Retrospective Cross-Sectional	Single Centered	Women With Severe Preeclampsia	Adverse Birth Out Come	549	Not reported	Not reported	Not reported	Not reported	Gestational Age, Platelets Count, Alanine Transaminase Birth Weight	Derivation	Logistic Regression
Griffin et,al, 2018 (7)	UK And Ireland	Prospective Cohort	Multi Centered	2nd And 3rd Trimester Pregnant	Adverse Birth Out Come	274	123	96	35%	9.6%	Nephrin, CPA-4a , Sflt-1, Endoglin, Plgfa , Plgf/S-Flt Ratioa , Plgf/Endoglin Ratioa , Plgfa + CPA-4â€ Plgf + Nephrin, Plgfa + Nephrin + CPA-4a	Derivation	Logistic Regression
Karlsen et al., 2015 (113)	Norway	Prospective Longitudinal	Single Centered	Singleton Pregnancy 24-Weeks	Adverse Birth Out Come	211	Not reported	89	42.2%	Not reported	Size And Conditional Growth Centiles	Derivation	Logistic Regression
Lagendijk et al., 2020 (114)	Netherland	Cluster Randomized Controlled Trial (C-RCT)	Multi Centered	Women Who Originally Participated In The Intervention Arm Of The C-RCT Of Rotterdam Reproductive Risk Reduction(R4U) Score Card	Adverse Birth Outcome	N/A	1752	282	16%	56.4	Insufficient Social Support, Single Mother, Experience Of inadequate social support, SGA Obstetric History and Preterm Baby Obstetric History	External Validation	Logistic Regression
Lean et al., 2021 (115)	UK	Prospective Cohort	Multi Centered	Pregnant Women At 28 Weeks	Adverse Perinatal Out Come	528	Not reported	108	20.46%	27	Total Antioxidant Capacity , 8-Isoprostane,Human Placental Lactogen ,Placental Growth Factor	Derivation	Logistic Regression
Mendoza et,al, 2021 (79)	Spain	prospective observational	Single centered	1st, 2nd and 3rd trimester pregnant women	Adverse perinatal out come	31	Not reported	14	45.2%	14	cervical pessary placement	Derivation	paired sample t-test

Table S6: Study characteristics prediction models for composite adverse birth outcome (continued)

Study	Model Development	Model Evaluation	Internal Validation	External Validation	Calibration (P Value Hosmer-Lemeshow Test Or Calibration Plot)	Model Discrimination (AUC With 95% CI)	Model Performances	Sensitivity With 95%CI	Specificity With 95%CI	Handling Of Mission Value	Method Of Handling Missing Value	Model Selection	Handling Of Continuous Data	How Is The Model Presented
Ngwenya. et al, 2021 (112)	The Model Develops From Maternal And Neonatal Character In Composite Outcome	AUC	Bootstrapping	No	Not reported	0.902(0.876,0.927)	No	Not reported	Not reported	Not reported	N/A	Stepwise Backward Selection	Categorized	Regression Coefficients
Griffin et,al, 2018 (7)	To Develop The Model Use Stepwise Logistic Regression For Bio Markers	AUC, Sensitivity, Specificity	Splitting	No	Not reported	0.83(NR, NR)	Yes	89.7% (91.7%, 94.7%)	58.7% (51.1%,66%)	Yes	Complete Case Analysis	Stepwise Selection (Forward Selection Followed By Series Of Backward Selection)	Kept Linear	Prediction Rule
Karlsen et al., 2015 (113)	Using Size And Growth Centiles Prediction Model Was Developed	Specificity Sensitivity	Not reported	No	Not reported	Not Reported	Yes	60%(49%, 69%)Using Size Centile, 39% (30%, 50%)Using Size And Growth Centile	78% (70%, 84%) For Size Centile, 94% (89%, 97%)For Size And Growth Centiles	No	Not reported	Stepwise Forward Selection	Kept Linear	Not reported
Lagendijk et al., 2020 (114)	N/A	AUC	N/A	Yes	With Calibration Plot	0.61 (0.56, 0.66)	NO	Not reported	Not reported	No	N/A	Stepwise Forward Selection	Categorized	Not reported
Lean et al., 2021 (115)	Model Developed Using Multivariable Logistic Regression Model	AUC	Not reported	Not reported	Not reported	Total Antioxidant Capacit;0.69(NR, NR) 8-Isoprostane = 0.66(NR,NR) Human Placental Lactogen =0.68(NR,NR	No	Not reported	Not reported	No	N/A	Stepwise Forward Selection	Categorized	Not reported

						),PlGF = 0.74(NR,NR)								
Mendoza et,al, 2021 (79)	The model develops from by cervical pessary placement	AUC	Not reported	Not reported	Not reported	0.88 (0.76,1.00)	Yes	100%	80%	Yes	Complete case analysis	Not reported	Not reported	Prediction rule

Reference

1. Hange U, Selvaraj R, Galani M, Letsholo K, editors. A data-mining model for predicting low birth weight with a high AUC. International Conference on Computer and Information Science; 2017: Springer.
2. Hassen HY, Gebreyesus SH, Endris BS, Roro MA, Van Geertruyden J-P. Development and validation of a risk score to predict low birthweight using characteristics of the mother: analysis from BUNMAP cohort in Ethiopia. Journal of clinical medicine. 2020;9(5):1587.
3. Hernández-Castro F, Berlanga-Garza A, Cruz-Gutiérrez MD, Soria-López JA, Villagómez-Martínez GE, Dávila-Escamilla IV. Prediction of low birth weight with hypoglycemia in glucose tolerance test. Revista de Saúde Pública. 2021;55.
4. Goyal NK, Hall ES, Greenberg JM, Kelly EA. Risk Prediction for Adverse Pregnancy Outcomes in a Medicaid Population. Journal of Womens Health. 2015;24(8):681-8.
5. Goyal NK, Hall ES, Greenberg JM, Kelly EA. Risk prediction for adverse pregnancy outcomes in a medicaid population. Journal of Women's Health. 2015;24(8):681-8.
6. O'Reilly-Green CP, Divon MY. Receiver operating characteristic curves of ultrasonographic estimates of fetal weight for prediction of fetal growth restriction in prolonged pregnancies. American journal of obstetrics and gynecology. 1999;181(5):1133-8.
7. Griffin M, Seed PT, Duckworth S, North R, Myers J, Mackillop L, et al. Predicting delivery of a small-for-gestational-age infant and adverse perinatal outcome in women with suspected pre-eclampsia. Ultrasound in Obstetrics & Gynecology. 2018;51(3):387-95.
8. Ryu A, Cho NJ, Kim YS, Lee EY. Predictive value of serum uric acid levels for adverse perinatal outcomes in preeclampsia. Medicine. 2019;98(18).
9. Schneuer FJ, Roberts CL, Ashton AW, Guilbert C, Tasevski V, Morris JM, et al. Angiopoietin 1 and 2 serum concentrations in first trimester of pregnancy as biomarkers of adverse pregnancy outcomes. American journal of obstetrics and gynecology. 2014;210(4):345. e1-. e9.
10. Bartnicki J, Casal D, Kreaden US, Saling E, Vetter K. Fetal fibronectin in vaginal specimens predicts preterm delivery and very-low-birth-weight infants. American journal of obstetrics and gynecology. 1996;174(3):971-4.
11. Cohen JL, Smilen KE, Bianco AT, Moshier EL, Ferrara LA, Stone JL. Predictive value of combined serum biomarkers for adverse pregnancy outcomes. European Journal of Obstetrics & Gynecology and Reproductive Biology. 2014;181:89-94.
12. Sinding M, Peters DA, Frøkjær JB, Christiansen OB, Petersen A, Uldbjerg N, et al. Prediction of low birth weight: Comparison of placental T2* estimated by MRI and uterine artery pulsatility index. Placenta. 2017;49:48-54.
13. Singh A, Arya S, Chellani H, Aggarwal K, Pandey R. Prediction model for low birth weight and its validation. The Indian Journal of Pediatrics. 2014;81(1):24-8.
14. Wu W, Zhang B, Li D, Yan M, Deng Q, Kang Y, et al. Development and validation of nomogram for prediction of low birth weight: a large-scale cross-sectional study in northwest China. The Journal of Maternal-Fetal & Neonatal Medicine. 2021:1-9.
15. Zahirzada A, Lavangnananda K, editors. Implementing predictive model for Low Birth Weight in Afghanistan. 2021 13th International Conference on Knowledge and Smart Technology (KST); 2021: IEEE.
16. Reeb KG, Graham AV, Zyzanski SJ, Kitson GC. Predicting low birthweight and complicated labor in urban black women: a biopsychosocial perspective. Social Science & Medicine. 1987;25(12):1321-7.
17. Rezaei M, Mehr AJ, Hashemian AH, Olang B, Rezaei K. Predicting factors of neonate birth weight. Der Pharmacia Lettre. 2016;8(1):457-63.
18. Owen J, Goldenberg RL, Davis RO, Kirk KA, Copper RL. Evaluation of a risk scoring system as a predictor of preterm birth in an indigent population. American journal of obstetrics and gynecology. 1990;163(3):873-9.
19. Carter J, Seed PT, Watson HA, David A, Sandall J, Shennan AH, et al. Development and validation of predictive models for QUiPP App v. 2: tool for predicting preterm birth in women with symptoms of threatened preterm labor. Ultrasound in obstetrics & gynecology. 2020;55(3):357-67.
20. Celik E, To M, Gajewska K, Smith G, Nicolaides K. Cervical length and obstetric history predict spontaneous preterm birth: development and validation of a model to provide individualized risk assessment. Ultrasound in Obstetrics and Gynecology: The Official Journal of the International Society of Ultrasound in Obstetrics and Gynecology. 2008;31(5):549-54.
21. Crane J, Hutchens D. Use of transvaginal ultrasonography to predict preterm birth in women with a history of preterm birth. Ultrasound in Obstetrics and Gynecology: The Official Journal of the International Society of Ultrasound in Obstetrics and Gynecology. 2008;32(5):640-5.
22. Damaso EL, Rolnik DL, Cavalli RdC, Quintana SM, Duarte G, da Silva Costa F, et al. Prediction of preterm birth by maternal characteristics and medical history in the Brazilian population. Journal of pregnancy. 2019;2019.

23. He J-R, Ramakrishnan R, Lai Y-M, Li W-D, Zhao X, Hu Y, et al. Predictions of preterm birth from early pregnancy characteristics: born in guangzhou cohort study. Journal of clinical medicine. 2018;7(8):185.
24. Hebbar S, Samjhana K. Role of mid-trimester transvaginal cervical ultrasound in prediction of preterm delivery. The Medical journal of Malaysia. 2006;61(3):307-11.
25. Hebert CL, Nattino G, Gabbe SG, Gabbe PT, Benedict J, Philips G, et al. Predictive Models for Very Preterm Birth: Developing a Point-of-Care Tool. American Journal of Perinatology. 2022;39(01):092-8.
26. Newman R, Goldenberg R, Iams J, Meis P, Mercer B, Moawad A, et al. Preterm prediction study: comparison of the cervical score and Bishop score for prediction of spontaneous preterm delivery. Obstetrics and gynecology. 2008;112(3):508.
27. Hellemans P, Gerris J, Verdonk P. Fetal fibronectin detection for prediction of preterm birth in low risk women. BJOG: An International Journal of Obstetrics & Gynaecology. 1995;102(3):207-12.
28. Fergus P, Cheung P, Hussain A, Al-Jumeily D, Dobbins C, Iram S. Prediction of preterm deliveries from EHG signals using machine learning. PloS one. 2013;8(10):e77154.
29. Jelliffe-Pawlowski LL, Rand L, Bedell B, Baer RJ, Oltman SP, Norton ME, et al. Correction: Prediction of preterm birth with and without preeclampsia using mid-pregnancy immune and growth-related molecular factors and maternal characteristics. Journal of Perinatology. 2018;38(7):946-.
30. Jung YJ, Kwon H, Shin J, Park Y, Heo S-J, Park HS, et al. The feasibility of cervical elastography in predicting preterm delivery in singleton pregnancy with short cervix following progesterone treatment. International journal of environmental research and public health. 2021;18(4):2026.
31. Nikolova T, Uotila J, Nikolova N, Bolotskikh VM, Borisova VY, Di Renzo GC. Prediction of spontaneous preterm delivery in women presenting with premature labor: a comparison of placenta alpha microglobulin-1, phosphorylated insulin-like growth factor binding protein-1, and cervical length. American journal of obstetrics and gynecology. 2018;219(6):610. e1-. e9.
32. Alleman BW, Smith AR, Byers HM, Bedell B, Ryckman KK, Murray JC, et al. A proposed method to predict preterm birth using clinical data, standard maternal serum screening, and cholesterol. American journal of obstetrics and gynecology. 2013;208(6):472. e1-. e11.
33. Andersen HF, Nugent CE, Wanty SD, Hayashi RH. Prediction of risk for preterm delivery by ultrasonographic measurement of cervical length. American journal of obstetrics and gynecology. 1990;163(3):859-67.
34. Grobman WA, Lai Y, Iams JD, Reddy UM, Mercer BM, Saade G, et al. Prediction of spontaneous preterm birth among nulliparous women with a short cervix. Journal of Ultrasound in Medicine. 2016;35(6):1293-7.
35. Park S, Oh D, Heo H, Lee G, Kim SM, Ansari A, et al. Prediction of preterm birth based on machine learning using bacterial risk score in cervicovaginal fluid. American Journal of Reproductive Immunology. 2021;86(3):e13435.
36. Pasquini L, Sisti G, Nasioudis D, Kanninen T, Sorbi F, Fambrini M, et al. Ability of cervical length to predict spontaneous preterm delivery in twin pregnancies using the receiver-operating characteristic curve analysis and an a priori cut-off value. Journal of Obstetrics and Gynaecology. 2017;37(5):562-5.
37. Posthumus A, Birnie E, van Veen M, Steegers E, Bonsel G. An antenatal prediction model for adverse birth outcomes in an urban population: the contribution of medical and non-medical risks. Midwifery. 2016;38:78-86.
38. Dziadosz M, Bennett T-A, Dolin C, Honart AW, Pham A, Lee SS, et al. Uterocervical angle: a novel ultrasound screening tool to predict spontaneous preterm birth. American journal of obstetrics and gynecology. 2016;215(3):376. e1-. e7.
39. Prema N, Pushpalatha M. Machine learning approach for preterm birth prediction based on maternal chronic conditions. Emerging Research in Electronics, Computer Science and Technology: Springer; 2019. p. 581-8.
40. García-Blanco A, Diago V, De La Cruz VS, Hervás D, Cháfer-Pericás C, Vento M. Can stress biomarkers predict preterm birth in women with threatened preterm labor? Psychoneuroendocrinology. 2017;83:19-24.
41. Raba G, Kotarski J. Evaluation of risk factors can help to predict preterm delivery within 7 days in women hospitalized for threatened preterm labour. The Journal of Maternal-Fetal & Neonatal Medicine. 2016;29(19):3142-6.
42. Rafael TJ, Hoffman MK, Leiby BE, Berghella V. Gestational age of previous twin preterm birth as a predictor for subsequent singleton preterm birth. American journal of obstetrics and gynecology. 2012;206(2):156. e1-. e6.
43. Crane JM, Armson BA, Dodds L, Feinberg RF, Kennedy W, Kirkland SA. Risk scoring, fetal fibronectin, and bacterial vaginosis to predict preterm delivery. Obstetrics & Gynecology. 1999;93(4):517-22.
44. Baer RJ, McLemore MR, Adler N, Oltman SP, Chambers BD, Kuppermann M, et al. Pre-pregnancy or first-trimester risk scoring to identify women at high risk of preterm birth. European Journal of Obstetrics & Gynecology and Reproductive Biology. 2018;231:235-40.
45. Riboni F, Vitulo A, Dell'avanzo M, Plebani M, Battagliarin G, Paternoster D. Biochemical markers predicting pre-term delivery in symptomatic patients: phosphorylated insulin-like growth factor binding protein-1 and fetal fibronectin. Archives of gynecology and obstetrics. 2011;284(6):1325-9.
46. Baños N, Murillo-Bravo C, Julià C, Migliorelli F, Perez-Moreno A, Ríos J, et al. Mid-trimester sonographic cervical consistency index to predict spontaneous preterm birth in a low-risk population. Ultrasound in Obstetrics & Gynecology. 2018;51(5):629-36.
47. Riboni F, Vitulo A, Plebani M, Dell'avanzo M, Battagliarin G, Paternoster D. Combination of biochemical markers in predicting pre-term delivery. Archives of gynecology and obstetrics. 2012;285(1):61-6.
48. Kim MA, Lee BS, Park YW, Seo K. Serum markers for prediction of spontaneous preterm delivery in preterm labour. European journal of clinical investigation. 2011;41(7):773-80.

49. Baraldi E, Giordano G, Stocchero M, Moschino L, Zaramella P, Tran MR, et al. Untargeted metabolomic analysis of amniotic fluid in the prediction of preterm delivery and bronchopulmonary dysplasia. PLoS One. 2016;11(10):e0164211.
50. Ridout AE, Ibeto LA, Ross GN, Cook JR, Sykes L, David AL, et al. Cervical length and quantitative fetal fibronectin in the prediction of spontaneous preterm birth in asymptomatic women with congenital uterine anomaly. American Journal of Obstetrics and Gynecology. 2019;221(4):341. e1-. e9.
51. Gioan M, Fenollar F, Loundou A, Menard J-P, Blanc J, d'Ercole C, et al. Development of a nomogram for individual preterm birth risk evaluation. Journal of Gynecology Obstetrics and Human Reproduction. 2018;47(10):545-8.
52. Koivu A, Sairanen M. Predicting risk of stillbirth and preterm pregnancies with machine learning. Health information science and systems. 2020;8(1):1-12.
53. Barinov SV, Di Renzo GC, Belinina AA, Koliado OV, Remneva OV. Clinical and biochemical markers of spontaneous preterm birth in singleton and multiple pregnancies. The Journal of Maternal-Fetal & Neonatal Medicine. 2021:1-6.
54. Kuhrt K, Hezelgrave N, Foster C, Seed P, Shennan A. Development and validation of a tool incorporating quantitative fetal fibronectin to predict spontaneous preterm birth in symptomatic women. Ultrasound in Obstetrics & Gynecology. 2016;47(2):210-6.
55. Rohlfing AB, Nah G, Ryckman KK, Snyder BD, Kasarek D, Paynter RA, et al. Maternal cardiovascular disease risk factors as predictors of preterm birth in California: a case–control study. BMJ open. 2020;10(6):e034145.
56. Bastek JA, Sammel MD, Srinivas SK, McShea MA, Foreman MN, Elovitz MA, et al. Clinical prediction rules for preterm birth in patients presenting with preterm labor. Obstetrics & Gynecology. 2012;119(6):1119-28.
57. Saade GR, Boggess KA, Sullivan SA, Markenson GR, Iams JD, Coonrod DV, et al. Development and validation of a spontaneous preterm delivery predictor in asymptomatic women. American journal of obstetrics and gynecology. 2016;214(5):633. e1-. e24.
58. Lee KA, Chang MH, Park M-H, Park H, Ha EH, Park EA, et al. A model for prediction of spontaneous preterm birth in asymptomatic women. Journal of Women's Health. 2011;20(12):1825-31.
59. Arabi Belaghi R, Beyene J, McDonald SD. Prediction of preterm birth in nulliparous women using logistic regression and machine learning. PloS one. 2021;16(6):e0252025.
60. Schmitz T, Kayem G, Maillard F, Lebret MT, Cabrol D, Goffinet F. Selective use of sonographic cervical length measurement for predicting imminent preterm delivery in women with preterm labor and intact membranes. Ultrasound in Obstetrics and Gynecology: The Official Journal of the International Society of Ultrasound in Obstetrics and Gynecology. 2008;31(4):421-6.
61. Lee KJ, Yoo J, Kim Y-H, Kim SH, Kim SC, Kim YH, et al. The clinical usefulness of predictive models for preterm birth with potential benefits: A Korean Preterm collaborate network (KOPEN) registry-linked data-based cohort study. International journal of medical sciences. 2020;17(1):1.
62. Menon R, Bhat G, Saade GR, Spratt H. Multivariate adaptive regression splines analysis to predict biomarkers of spontaneous preterm birth. Acta obstetricia et gynecologica Scandinavica. 2014;93(4):382-91.
63. Lee S, Park J, Norwitz E, Oh S, Kim E, Kim S, et al. Mid-trimester amniotic fluid pro-inflammatory biomarkers predict the risk of spontaneous preterm delivery in twins: a retrospective cohort study. Journal of Perinatology. 2015;35(8):542-6.
64. Leow SM, Di Quinzio MK, Ng ZL, Grant C, Amitay T, Wei Y, et al. Preterm birth prediction in asymptomatic women at mid-gestation using a panel of novel protein biomarkers: the Prediction of PreTerm Labor (PPeTaL) study. American Journal of Obstetrics & Gynecology MFM. 2020;2(2):100084.
65. Leung T, Pang M, Leung T, Poon C, Wong S, Lau T. Cervical length at 18–22 weeks of gestation for prediction of spontaneous preterm delivery in Hong Kong Chinese women. Ultrasound in Obstetrics and Gynecology: The Official Journal of the International Society of Ultrasound in Obstetrics and Gynecology. 2005;26(7):713-7.
66. Lin Y, He F, Gao R, Liu T, Zhao K, He J, et al. A Clinical Nomogram For Predicting Preterm Birth in Women Who Conceived Through Assisted Reproductive Technology. 2021.
67. Liu Y, Zhu Y, Liu Y, Liu M, Zhang Y, Chen J, et al. Nomogram to predict the risk of preterm birth before 37 weeks and 34 weeks in pregnant women with a short cervix. The Journal of Maternal-Fetal & Neonatal Medicine. 2020:1-10.
68. van de Mheen L, Schuit E, Lim AC, Porath MM, Papatsonis D, Erwich JJ, et al. Prediction of preterm birth in multiple pregnancies: development of a multivariable model including cervical length measurement at 16 to 21 weeks' gestation. Journal of obstetrics and gynaecology Canada. 2014;36(4):309-19.
69. Yazici G, Yildiz A, Tiras MB, Arslan M, Kanik A, Oz U. Comparison of transperineal and transvaginal sonography in predicting preterm delivery. Journal of Clinical Ultrasound. 2004;32(5):225-30.
70. Mueller-Heubach E, Guzick DS. Evaluation of risk scoring in a preterm birth prevention study of indigent patients. American Journal of Obstetrics and Gynecology. 1989;160(4):829-37.
71. Mortensen OA, Franklin J, Lofstrand T, Svanberg B. Prediction of preterm birth. Acta Obstetricia et Gynecologica Scandinavica. 1987;66(6):507-12.
72. Morken NH, Kaellen K, Jacobsson B. Predicting risk of spontaneous preterm delivery in women with a singleton pregnancy. Paediatric and perinatal epidemiology. 2014;28(1):11-22.
73. Zhang J, Pan M, Zhan W, Zheng L, Jiang X, Xue X. Two-stage nomogram models in mid-gestation for predicting the risk of spontaneous preterm birth in twin pregnancy. Archives of Gynecology and Obstetrics. 2021;303(6):1439-49.
74. Macdonald-Wallis C, Silverwood RJ, De Stavola BL, Inskip H, Cooper C, Godfrey KM, et al. Antenatal blood pressure for prediction of pre-eclampsia, preterm birth, and small for gestational age babies: development and validation in two general population cohorts. Bmj. 2015;351.
75. Mailath-Pokorny M, Polterauer S, Kohl M, Kueronyai V, Worda K, Heinze G, et al. Individualized assessment of preterm birth risk using two modified prediction models. European Journal of Obstetrics & Gynecology and Reproductive Biology. 2015;186:42-8.
76. McLean M, Bisits A, Davies J, Walters W, Hackshaw A, De Voss K, et al. Predicting risk of preterm delivery by second-trimester measurement of maternal plasma corticotropin-releasing hormone and α-fetoprotein concentrations. American journal of obstetrics and gynecology. 1999;181(1):207-15.

77. Watson H, Seed P, Carter J, Hezelgrave N, Kuhrt K, Tribe R. Development and validation of the predictive models for the QUiPP App vol 2: a tool for predicting preterm birth in high-risk asymptomatic women. Ultrasou Obstet Gynecol. 2019.
78. Mehta-Lee SS, Palma A, Bernstein PS, Lounsbury D, Schlecht NF. A preconception nomogram to predict preterm delivery. Maternal and child health journal. 2017;21(1):118-27.
79. Mendoza M, Maiz N, Garcia-Ruiz I, Vargas M, Rodo C, Goya M, et al. Prediction of preterm birth and adverse perinatal outcomes after cervical pessary placement in singleton pregnancies with short cervical length. The Journal of Maternal-Fetal & Neonatal Medicine. 2021;34(19):3147-53.
80. Zhu Y-z, Peng G-q, Tian G-x, Qu X-l, Xiao S-y. New model for predicting preterm delivery during the second trimester of pregnancy. Scientific reports. 2017;7(1):1-9.
81. Radhouane A, Nadia BJ, Imen K, Cheour M, Ines B, Feirouz A, et al. Ultrasound cervical length in predicting preterm birth: Prospective study. Australasian Medical Journal (Online). 2017;10(8):647.
82. Bachkangi P, Taylor A, Bari M, Maccarrone M, Konje JC. Prediction of preterm labour from a single blood test: The role of the endocannabinoid system in predicting preterm birth in high-risk women. European Journal of Obstetrics & Gynecology and Reproductive Biology. 2019;243:1-6.
83. Szymusik I, Wegrzyn P, Marianowski P, Dziadecki W, Bablok L, Wielgos M. Is mid-gestational cervical length measurement as sensitive prediction factor of preterm delivery in IVF as in spontaneous singleton pregnancies? Neuroendocrinology Letters. 2011;32(4).
84. Schaaf JM, Ravelli AC, Mol BWJ, Abu-Hanna A. Development of a prognostic model for predicting spontaneous singleton preterm birth. European Journal of Obstetrics & Gynecology and Reproductive Biology. 2012;164(2):150-5.
85. Akolekar R, Tokunaka M, Ortega N, Syngelaki A, Nicolaides KH. Prediction of stillbirth from maternal factors, fetal biometry and uterine artery Doppler at 19–24 weeks. Ultrasound in Obstetrics & Gynecology. 2016;48(5):624-30.
86. Akolekar R, Machuca M, Mendes M, Paschos V, Nicolaides K. Placental growth factor in prediction of stillbirths at 11–13 weeks. Ultrasound Obstet Gynecol. 2016;48:618-23.
87. Åmark H, Westgren M, Persson M. Prediction of stillbirth in women with overweight or obesity—A register-based cohort study. PloS one. 2018;13(11):e0206940.
88. Aupont JE, Akolekar R, Illian A, Neonakis S, Nicolaides KH. Prediction of stillbirth from placental growth factor at 19–24 weeks. Ultrasound in Obstetrics & Gynecology. 2016;48(5):631-5.
89. Bahado-Singh RO, Syngelaki A, Mandal R, Han B, Li L, Bjorndahl TC, et al. First-trimester metabolomic prediction of stillbirth. The Journal of Maternal-Fetal & Neonatal Medicine. 2019;32(20):3435-41.
90. Mastrodima S, Akolekar R, Yerlikaya G, Tzelepis T, Nicolaides KH. Prediction of stillbirth from biochemical and biophysical markers at 11–13 weeks. Ultrasound in obstetrics & gynecology. 2016;48(5):613-7.
91. Kim M-A, Han GH, Kim Y-H. Prediction of small-for-gestational age by fetal growth rate according to gestational age. PloS one. 2019;14(4):e0215737.
92. Antunes IR, Lobo GAR, Júnior EA, da Silva Pares DB. Predictive values of clinical parameters and biophysical and biochemical markers in the first trimester for the detection of small-for-gestational age fetuses. Journal of Perinatal Medicine. 2021;49(1):73-9.
93. Poon LC, Lesmes C, Gallo DM, Akolekar R, Nicolaides KH. Prediction of small-for-gestational-age neonates: screening by biophysical and biochemical markers at 19–24 weeks. Ultrasound in Obstetrics & Gynecology. 2015;46(4):437-45.
94. Bækgaard Thorsen LH, Bjørkholt Andersen L, Birukov A, Lykkedegn S, Dechend R, Stener Jørgensen J, et al. Prediction of birth weight small for gestational age with and without preeclampsia by angiogenic markers: an Odense Child Cohort study. The Journal of Maternal-Fetal & Neonatal Medicine. 2020;33(8):1377-84.
95. Kato DMP, Lorusso L, Bruns RF, Pinhat EC, Dalla Costa NRA, Pletsch L, et al. Performance of a local reference curve for predicting small for gestational age fetuses in pregnant women with HIV/AIDS. Journal of Clinical Ultrasound. 2021;49(4):322-7.
96. Quant HS, Sammel MD, Parry S, Schwartz N. Second-trimester 3-dimensional placental sonography as a predictor of small-for-gestational-age birth weight. Journal of Ultrasound in Medicine. 2016;35(8):1693-702.
97. Quinton A, Cook C, Peek M. The prediction of the small for gestational age fetus with the head circumference to abdominal circumference (HC/AC) ratio: a new look at an old measurement. Sonography. 2015;2(2):27-31.
98. Leavitt K, Odibo L, Nwabuobi C, Tuuli MG, Odibo A. The value of introducing cerebroplacental ratio (CPR) versus umbilical artery (UA) Doppler alone for the prediction of neonatal small for gestational age (SGA) and short-term adverse outcomes. The Journal of Maternal-Fetal & Neonatal Medicine. 2021;34(10):1565-9.
99. Schwartz N, Sammel MD, Leite R, Parry S. First-trimester placental ultrasound and maternal serum markers as predictors of small-for-gestational-age infants. American journal of obstetrics and gynecology. 2014;211(3):253. e1-. e8.
100. Schwartz N, Wang E, Parry S. Two-dimensional sonographic placental measurements in the prediction of small-for-gestational-age infants. Ultrasound in obstetrics & gynecology. 2012;40(6):674-9.
101. Lesmes C, Gallo DM, Gonzalez R, Poon LC, Nicolaides KH. Prediction of small-for-gestational-age neonates: screening by maternal serum biochemical markers at 19–24 weeks. Ultrasound in Obstetrics & Gynecology. 2015;46(3):341-9.
102. Seravalli V, Block-Abraham DM, Turan OM, Doyle LE, Blitzer MG, Baschat AA. Second-trimester prediction of delivery of a small-for-gestational-age neonate: integrating sequential Doppler information, fetal biometry, and maternal characteristics. Prenatal diagnosis. 2014;34(11):1037-43.
103. Lesmes C, Gallo D, Panaiotova J, Poon L, Nicolaides K. Prediction of small-for-gestational-age neonates: screening by fetal biometry at 19–24 weeks. Ultrasound in Obstetrics & Gynecology. 2015;46(2):198-207.
104. Lesmes C, Gallo DM, Saiid Y, Poon LC, Nicolaides KH. Prediction of small-for-gestational-age neonates: screening by uterine artery Doppler and mean arterial pressure at 19–24 weeks. Ultrasound in Obstetrics & Gynecology. 2015;46(3):332-40.

105. Souka A, Papastefanou I, Pilalis A, Michalitsi V, Kassanos D. Performance of third-trimester ultrasound for prediction of small-for-gestational-age neonates and evaluation of contingency screening policies. Ultrasound in obstetrics & gynecology. 2012;39(5):535-42.
106. Souka AP, Papastefanou I, Pilalis A, Michalitsi V, Panagopoulos P, Kassanos D. Performance of the ultrasound examination in the early and late third trimester for the prediction of birth weight deviations. Prenatal Diagnosis. 2013;33(10):915-20.
107. MacDonald TM, Tran C, Kaitu'u-Lino TuJ, Brennecke SP, Hiscock RJ, Hui L, et al. Assessing the sensitivity of placental growth factor and soluble fms-like tyrosine kinase 1 at 36 weeks' gestation to predict small-for-gestational-age infants or late-onset preeclampsia: a prospective nested case-control study. BMC pregnancy and childbirth. 2018;18(1):1-12.
108. Ye C, Kobayashi H, Katagiri S, Miyasaka N, Takeuchi Y, Kuraji R, et al. The relationship between the anti-Porphyromonas gingivalis immunoglobulin G subclass antibody and small for gestational age delivery: a longitudinal study in pregnant Japanese women. International Dental Journal. 2020;70(4):296-302.
109. McCowan LM, Thompson JM, Taylor RS, Baker PN, North RA, Poston L, et al. Prediction of small for gestational age infants in healthy nulliparous women using clinical and ultrasound risk factors combined with early pregnancy biomarkers. PLoS One. 2017;12(1):e0169311.
110. Wen SW, Tan H, Yang Q, Walker M. Prediction of small for gestational age by logistic regression in twins. Australian and New Zealand journal of obstetrics and gynaecology. 2005;45(5):399-404.
111. Rosendahl H, Kivinen S. Detection of small for gestational age fetuses by the combination of clinical risk factors and ultrasonography. European Journal of Obstetrics & Gynecology and Reproductive Biology. 1991;39(1):7-11.
112. Ngwenya S, Jones B, Mwembe D, Nare H, Heazell AEP. Development and validation of risk prediction models for adverse maternal and neonatal outcomes in severe preeclampsia in a low-resource setting, Mpilo Central Hospital, Bulawayo, Zimbabwe. Pregnancy Hypertension. 2021;23:18-26.
113. Karlsen H, Johnsen S, Rasmussen S, Kiserud T. Prediction of adverse neonatal outcomes using size centiles and conditional growth centiles. Ultrasound in obstetrics & gynecology: the official journal of the International Society of Ultrasound in Obstetrics and Gynecology. 2015.
114. Lagendijk J, Steyerberg EW, Daalderop LA, Been JV, Steegers EA, Posthumus AG. Validation of a prognostic model for adverse perinatal health outcomes. Scientific Reports. 2020;10(1):1-7.
115. Lean SC, Jones RL, Roberts SA, Heazell AE. A prospective cohort study providing insights for markers of adverse pregnancy outcome in older mothers. BMC pregnancy and childbirth. 2021;21(1):1-17.
